# Potential effect of *Wolbachia* on virus restriction in the spider mite *T. truncatus*

**DOI:** 10.3389/fmicb.2025.1570606

**Published:** 2025-05-29

**Authors:** Lucas Yago Melo Ferreira, João Pedro Nunes Santos, David Gabriel do Nascimento Souza, Lixsy Celeste Bernardez Orellana, Sabrina Ferreira de Santana, Anderson Gonçalves Sousa, Paula Luize Camargos Fonseca, Amanda Gabrielly Santana Silva, Vinicius Castro Santos, Isaque João da Silva de Faria, Roenick Proveti Olmo, Luis Gustavo Carvalho Pacheco, Marcio Gilberto Cardoso Costa, Carlos Priminho Pirovani, Anibal Ramadan Oliveira, Eric Roberto Guimarães Rocha Aguiar

**Affiliations:** ^1^Center of Biotechnology and Genetics, Department of Biological Sciences, Universidade Estadual de Santa Cruz, Ilhéus, Bahia, Brazil; ^2^Department of Genetics, Instituto de Ciências Biológicas, Universidade Federal de Minas Gerais, Belo Horizonte, Minas Gerais, Brazil; ^3^Department of Biochemistry and Immunology, Instituto de Ciências Biológicas, Universidade Federal de Minas Gerais, Belo Horizonte, Minas Gerais, Brazil; ^4^CNRS UPR9022, Inserm U1257, Institut de Biologie Moléculaire et Cellulaire, Strasbourg, France; ^5^Department of Biotechnology, Institute of Health Sciences, Universidade Federal da Bahia (UFBA), Salvador, Bahia, Brazil; ^6^Laboratory of Entomology, Department of Biological Science, Universidade Estadual de Santa Cruz, Ilhéus, Bahia, Brazil; ^7^Department of Engineering and Computing (DEC), Universidade Estadual de Santa Cruz, Ilhéus, Bahia, Brazil

**Keywords:** *Wolbachia*, virome, Tetranychidae, transcriptomics, bioinformatics

## Abstract

The mite *T. truncatus* is a significant agricultural pest and may serve as a potential vector for viral transmission. However, the virome of *T. truncatus* remains understudied. Through metatranscriptomic analyses of publicly available data, we uncovered a diverse range of viruses associated with the spider mite, including crop-infecting pathogenic species such as *Potato virus Y* and *Cherry virus A*, and fourteen previously unknown viruses across several families (e.g., *Virgaviridae*, *Dicistroviridae*, *Kitaviridae*, *Betaflexiviridae*, and *Nudiviridae*). Taking advantage of mite samples under different conditions, we also assessed the impact of biotic (*Wolbachia* and *Spiroplasma* infection) and abiotic stresses (pesticide exposure and temperature stress) on the *T. truncatus* virome. Interestingly, *Wolbachia* appeared to restrict viral infections in *T. truncatus* by reducing viral diversity and abundance, with a pronounced effect on dicistroviruses. Surprisingly, a similar effect also observed with *Spiroplasma*. However, the viral restriction phenotype vanishes in co-infected mites. Transcriptomics analysis of singly-infected mites revealed upregulation of piRNA and autophagy-related genes, while lipid metabolism processes-related genes were downregulated, indicating an endosymbiont-sharing mechanisms of viral interference. Although the impact of abiotic stressors on the virome was not statistically significant, *Potato virus Y* and TtDV-2 viruses were absent in abamectin-exposed mites, suggesting a potential reduction in the viral diversity, while heat-stressed mites exhibited slightly higher viral diversity compared to those raised at regular temperatures. Overall, our work provides a detailed analysis of the *T. truncatus* virome, shedding light on how endosymbionts and environmental factors shape viral dynamics and offering potential insights for pest management strategies.

## Introduction

Mites (Arachnida: Acari) are an important group of arthropods that play a significant role as agricultural pests. They cause direct damage to plants by feeding on tissues or indirectly by acting as vectors for viruses, which can severely affect plant health, reducing vigor or even causing plant death in extreme cases (Hoy, 2011; Sarwar, 2020). Among these, the *Tetranychidae* family, which includes spider mites, is particularly notable. These mites infest over 4,000 plant species and are recognized as major agricultural pests. One such species, *T. truncatus*, commonly known as the red spider mite, is a significant pest that infests approximately 104 plant species, including major crops such as bean, papaya, corn, soy, cotton, maize, and cassava. Despite its widespread agricultural impact, including crop damage that can reach up to 73% in some cases (Guo et al., 2013; Vacante, 2016), there is no direct evidence linking *T. truncatus* to the transmission of plant pathogens.

In contrast, several other species within the *Tetranychidae* family have been identified as vectors of plant viruses. For example, *Petrobia latens* has been shown to transmit Barley Yellow Mosaic Virus (BaYSMV), primarily vectored by *Polymyxa graminis* (Adams et al., 1988; Smidansky and Carroll, 1996). *Tetranychus urticae*, the two-spotted spider mite, is linked to the transmission of Potato Virus Y (PVY), although aphids are the primary vectors for this virus (Schulz, 1963; Gray and Power, 2018). Additionally, *T. urticae* has been associated with the transmission of several other viruses, including *Tobacco Ringspot Virus* (TRSV), which is typically spread by nematodes (Yang et al., 2020), *Tobacco Mosaic Virus* (TMV), primarily transmitted through mechanical means (Heinlein, 2002), *Southern Bean Mosaic Virus* (SBMV), transmitted by beetles (Kopek and Scott, 1983), and *Cotton Leaf Curl Virus* (CLCuV), which is spread by whiteflies (Briddon and Markham, 2000).

In insects and other arthropods that vector viruses, endosymbiotic bacteria can profoundly influence both host fitness and viral dynamics. The α-proteobacterium *Wolbachia* infects up to 76% of insect species yet inhibits viral replication not exclusively through cytoplasmic incompatibility (CI) itself—which manipulates host reproduction to promote symbiont spread—but via immune priming and resource competition mechanisms. These effects have raised interest in using *Wolbachia* to control virus transmission, particularly after its success in mosquitoes, which has led to further exploration in other arthropods, including spider mites (Min and Benzer, 1997; Weeks et al., 2002; Hoffmann et al., 2011; Zhu et al., 2021). Endosymbionts, including *Rickettsia* spp. (Kliot et al., 2014) and Arsenophonus (Kaur et al., 2022), play crucial roles in viral dynamics. For example, *Rickettsia* facilitates viral transmission in Bemisia tabaci, while Rosenbergiella reduces viral transmission in Aedes mosquitoes (Zhang et al., 2024). Similarly, *Spiroplasma*, known for its protective roles against bacterial infections, parasitism, and nematode damage in species like Drosophila melanogaster and aphids (Hamilton et al., 2016; Ballinger and Perlman, 2019; Hrdina et al., 2024), has shown promise in reducing viral transmission. Although no direct evidence of *Spiroplasma*’s antiviral properties is available, certain strains produce ribosome-inactivating proteins (RIPs) that target rRNA. Plants-derived RIPs have demonstrated antiviral activity in several viruses, including HIV (Kaur et al., 2011), DENV, and CHIKV (Citores et al., 2021). Additionally, *Spiroplasma* strains are known to induce cytoplasmic incompatibility and may engage in resource competition mechanisms akin to those observed in *Wolbachia*, potentially contributing to phenotypes associated with reduced viral loads (Sinkins, 2004; Werren et al., 2008). These endosymbionts have substantial implications for pest control and virus management in agriculture (Werren et al., 2008; Moreira et al., 2009).

Beyond the biological interactions between endosymbionts and viruses, environmental factors, particularly temperature, is known to shape symbiont densities, mite development, and viral dynamics. Fluctuations in temperature modulate endosymbiont replication, alter the pace of mite growth and reproduction, and influence viral replication rates, microbiome composition, host susceptibility, and immune responsiveness—collectively determining the efficiency of virus transmission (Van Opijnen and Breeuwer, 1999; Lowen et al., 2007; Kilpatrick et al., 2008). Furthermore, the use of pesticides, such as abamectin, plays a crucial role in modern pest control strategies (Kole et al., 2019; Kumar et al., n.d.). While effective in mitigating pest populations, abamectin also impacts arthropod survival, and, potentially, alters viral load (Varghese et al., 2016; Khodayari and Hamedi, 2022).

Our study investigates the virome of the spider mite *T. truncatus*, focusing on the impact of biotic and abiotic stresses on its viral diversity and abundance. Using 39 publicly available high-throughput RNA sequencing (RNA-seq) libraries from diverse locations and conditions—including abamectin exposure, variable temperature, and *Wolbachia* and/or *Spiroplasma* infections—we identified both known and novel virus species. We also assessed how these environmental and biological factors influenced the mite’s virome.

## Materials and methods

### RNA libraries

Publicly available *T. truncatus* RNAseq libraries were obtained from the Sequence Read Archive (SRA) database at NCBI. A total of 39 libraries, representing diverse sampling conditions and geographical origins across five distinct Bioprojects, were selected for this study. Among these, 25 libraries were used as input for virus discovery while 14 were used as biological replicates to access independent RNA levels. Of note, each of the libraries were analyzed individually according to the analysis objective. Overall, these libraries were constructed from total RNA of *T. truncatus* individuals, including libraries from mites (i) reared on different host plants (soybean, eggplant, and tomato) - PRJNA636216, (ii) reared in heat-stressed environments - PRJNA881040, (iii) infected or co-infected with *Wolbachia pipientis* and/or *Spiroplasma ixodetis -* PRJNA717652 and PRJNA644209, and (iv) exposed or not exposed to the acaricide abamectin - PRJNA475023. Detailed description of each individual library used in the work is available in Supplementary File 1.

### Identification of endogenous viral elements (EVEs)

To provide disambiguation between possible Endogenous Viral Elements (EVEs) and exogenous virus species circulating in *T. truncatus*, we screened the reference genome GCA_028476895.1 (Chen et al., 2023) using the pipeline previously described in Aguiar et al. (2020).

### Metaviromic Analyses

*De novo* virus discovery in RNAseq libraries was performed as described in Espinal et al. (2023). Briefly, for each library quality control of sequenced reads was assessed using FastQC (version 0.74 + galaxy0) (Wingett and Andrews, 2018) and low-quality reads (Phred < 20) and adaptor sequences were removed using Trimmomatic (Galaxy Version 0.38.1) (Bolger et al., 2014). Trimmed reads were aligned against the host genome using Bowtie2 (version 2.5.0 + galaxy0) (Langmead and Salzberg, 2012) with default settings to identify host-related reads. Unaligned reads were assembled in parallel using different assembly tools including Trinity (version 2.15.1 + galaxy0) (Grabherr et al., 2011), SPAdes (version 3.15.4 + galaxy1) (Prjibelski et al., 2014), rnaviralSPAdes (version 3.15.4 + galaxy2) (Antipov et al., 2016), metaviralSPAdes (Antipov et al., 2019), Megahit (Li et al., 2015), and OASIS (Schulz et al., 2012). To identify potential virus-derived contigs, sequence similarity search was performed using Diamond (2.0.15 + galaxy0) (Buchfink et al., 2015) with BlastX mode, utilizing the NCBI Viral Refseq database (release 218) as reference. These analyses were conducted using the Galaxy Australia platform (Afgan et al., 2018).

### Manual curation of contigs annotated as viral genomes

Non-retroviral sequences were filtered based on a minimum size threshold of 500 nucleotides. Filtered sequences were manually examined through online version of BLAST against Nucleotide (NT) and Protein (NR) databases (release 04/2024). The ORFfinder (Rombel et al., 2002) tool was used to predict open reading frames (ORFs) within contigs and InterPro (Blum et al., 2021), Hmmer (Finn et al., 2011, 2015; Potter et al., 2018), and CDblast (Marchler-Bauer and Bryant, 2004) to identify conserved protein domains. An overview of BLAST best hits at nucleotide and amino acid levels for each virus-derived contig can be visualized in the Supplementary File 2.

### Phylogenetic analysis

Sequences showing similarity to genes encoding for polymerases or polyproteins were used to build phylogenetic trees. MAFFT (Katoh and Standley, 2013) was used for global alignment using standard parameters. ModelTest (Posada and Crandall, 1998) was used to identify the best evolutionary model for the dataset and a maximum likelihood phylogenetic tree was inferred using 1,000 bootstrap replicates. The CIPRES Science Gateway (Miller et al., 2010) was used for phylogenetic tree construction.

### RNA abundance of viral segments

The tool Salmon (version 1.5.1 + galaxy0) (Patro et al., 2017) was used to assess the abundance of virus-derived sequences for each library. The host mitochondrial ribosomal protein S11 (rps) protein and nuclear calmodulin-1 (cal) genes were selected as constitutive endogenous genes for comparison with virus abundance. An overview of the abundance of the viral sequences can be visualized in Supplementary File 3.

### Diversity and batch effect analysis

Assessment of alpha diversity per library was performed using the package VEGAN on R (Oksanen et al., 2008) utilizing the richness (Fisher et al., 1943) to unravel the virus diversity within the conditions. In order to address potential confounding factors and ensure the robustness of our dataset, we performed batch effect analysis using RUVSeq (Risso et al., 2014).

### Improving the annotation of *T. truncatus* genome

To improve the current *T. truncatus* gene annotation, we performed a comprehensive gene reannotation utilizing the Blast2GO v3.3.2 (Conesa et al., 2005) pipeline (November/2023). The process began with sequence alignment via Diamond/Blast v2.1.8, followed by domain search using InterProScan v5.64-96.0. Orthologous groups were identified through eggNOG-mapper v2.1.12 (Cantalapiedra et al., 2021), after which GO mapping was performed. Finally, the Blast2GO functional annotation algorithm was employed to complete the reannotation. This pipeline ensured the inclusion of genes from the Arthropoda phylum, resulting in the annotation of over 400 additional genes compared to the previously available annotation. The novel annotation is available in the Supplementary File 4.

### Ortholog inference, differential gene expression, and gene set enrichment analysis

In order to have a functional annotation for the *T. truncatus* genome (GCA_028476895.1), we deployed the tool OrthoFinder (Emms and Kelly, 2019) v2.5.5.2 with default parameters to perform ortholog inference, using as reference the well described fly model Drosophila melanogaster (genome release FB2024_01) - available in Supplementary File 5. Subsequently, to unveil potential variations in gene expression across different conditions, we performed differential gene expression analysis utilizing DESeq2 (Love et al., 2014). This analysis was conducted exclusively on libraries from the *Wolbachia* and/or *Spiroplasma* bioprojects (PRJNA717652 and PRJNA644209), which originate from mite populations sharing the same genetic background and experimental conditions. Gene expression differences were assessed by comparing control samples to those exposed to stressors. Genes were considered significantly differentially expressed when having a fold change > 2 log_2_ and *p*-value < 0.05. Gene Set Enrichment Analysis (GSEA) – ClusterProfiler (Yu et al., 2012) (version 3.8) (Shi and Walker, 2007) was used to elucidate enriched pathways using D. melanogaster gene sets (version 3.13.0) as model obtained from Bioconductor annotation packages (Gentleman et al., 2004).

## Results

### Virome of spider mites

**FIGURE 1:**
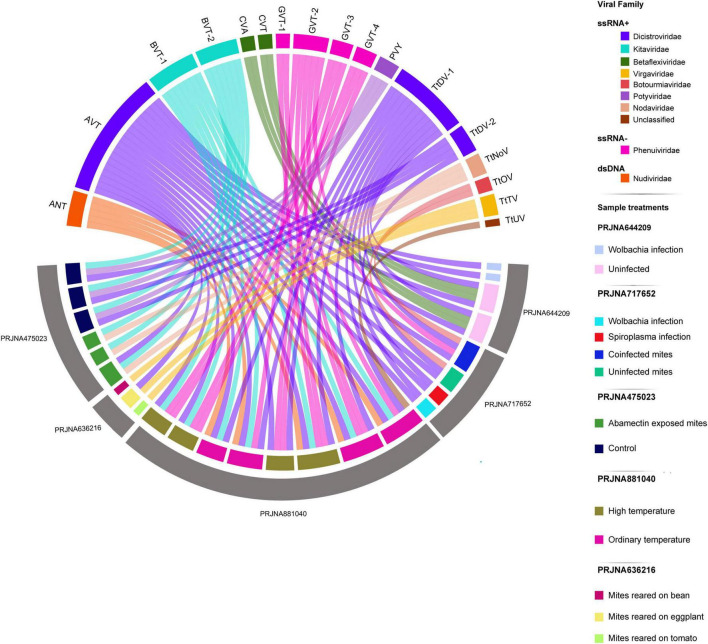
Overview of viruses identified in *T. truncatus* samples classified by Bioproject identifiers, conditions, genome composition and viral family. The diagram depicts the flow of viral contigs across different Bioprojects and libraries. The upward-facing arcs represent viral contigs, colored according to their viral family. The first downward-facing arc corresponds to libraries, colored by treatment conditions, while the second downward-facing arc reflects the Bioprojects. Contigs matching (+)ssRNA viruses composed 68.75% of the total and included Blunervirus truncatus 1 (BVT-1), Blunervirus truncatus 2 (BVT-2), *Cherry virus A* (CVA), Citrivirus truncatum (CVT), Aparavirus truncatus (AVT), *Potato virus Y* (PVY), *T. truncatus*-associated dicistro-like virus 1 (TtDV-1), *T. truncatus*-associated dicistro-like virus 2 (TtDV-2), *T. truncatus*-associated noda-like virus (TtNoV), *T. truncatus*-associated ourmia-like virus (TtOV), *T. truncatus*-associated tobamo-like virus (TtTV), and *Acyrthosiphon pisum virus* (AcPV). Contigs matching (–)ssRNA represented 25.00% and included Goukovirus truncatum 1 (GVT-1), Goukovirus truncatum 2 (GVT-2), Goukovirus truncatum 3 (GVT-3), and Goukovirus truncatum 4 (GVT-4). Contigs annotated as dsDNA viruses represented 6.25% and included the Alphanudivirus truncatus (ANT).

To characterize the virome of *T. truncatus*, we retrieved publicly available RNAseq libraries covering a wide range of biological and environmental conditions and performed *Sabrina Ferreira Isaque João da Silva de Faria Santana novo* virus identification using an unbiased virus discovery pipeline (Espinal et al., 2023). Our analysis revealed at least 30 potential virus-derived assembled contigs belonging to known and new virus species (Figure 1 and Supplementary File 2). Among these, three contigs showed high sequence similarity to the genome of known viruses and coded for similar protein domains as their BLAST best hits. Among them, one contig showed 99.64% nucleotide identity and 99.77% amino acid identity to *Potato virus Y*, another contig had 99.47% nucleotide identity and 99.70% amino acid identity with *Cherry virus A*, while the last contig had 99.63% nucleotide identity and 99.81% amino acid identity with *Acyrthosiphon pisum virus* (Supplementary Figure 1). Ten additional contigs showed sequence similarity above 50% at the amino acid level and similar conserved domains to positive single-strand RNA ((+) ssRNA) viruses (Figure 1 and Supplementary File 2). Phylogenetic analyses grouped three putative contigs as viruses belonging to the *Dicistroviridae* family. The remaining contigs showed sequence similarity to the *Kitaviridae*, *Betaflexiviridae*, *Virgaviridae*, *Botourmiaviridae*, and *Nodaviridae* families (Figure 1 and Supplementary File 2). Among these, seven contigs contained polyproteins with start and stop codons, two contigs encoded for RdRp and capsid segments similar to nodaviruses, and one contig resemble a fragment of a narnavirus RdRp (Figure 1 and Supplementary Figures 2–7, 8A–F, 9A–C, respectively). Four contigs exhibited sequence similarity above 30% and their ORFs contained conserved domains commonly present in the negative single-stranded RNA viral family *Phenuiviridae*. These contigs encoded for three incomplete and one complete RdRp protein (Supplementary Figures 9D–G, 10). The remaining 13 contigs matched both in amino acid sequence similarity and conserved domains to core genes of the dsDNA family *Nudiviridae* (Supplementary Figures 11, 12).

One major issue in virus discovery works is the presence of endogenous viral elements (EVEs) that can mislead the identification of exogenous viruses in metagenomics data. To avoid this issue, we performed de novo identification of EVEs on the *T. truncatus* genome. We identified three endogenized sequences sharing similarities with viral proteins of the Chuviridae and Rhabdoviridae families (see Supplementary File 6 for details). Assembled contigs from RNAseq libraries matching any of these potential EVEs with sequence similarity above 70% and similar length were discarded from our analyses.

### Prevalence and widespread of *T. truncatus*-associated viruses

**FIGURE 2:**
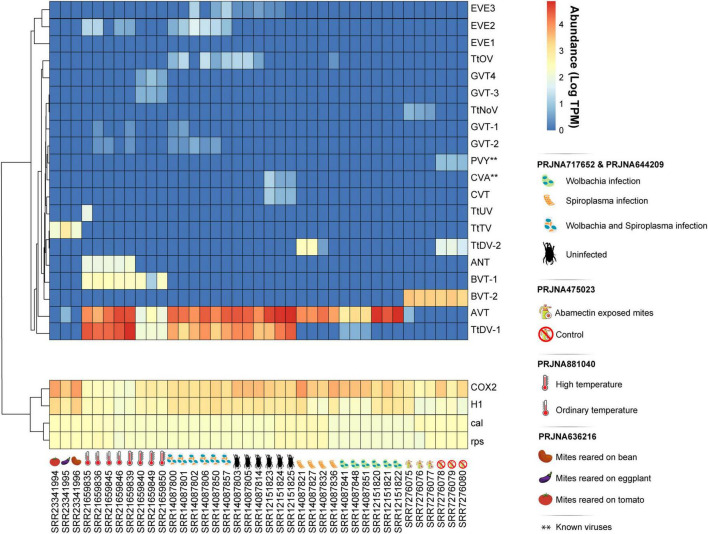
Abundance of identified viral sequences associated to *T. truncatus*. Rows were clustered based on Pearson correlation to group sequences with similar abundance calculated as transcripts per million (TPM). Samples are separated by Bioproject with respect to their treatment conditions. Bioprojects PRJNA717652 and PRJNA644209 include libraries from populations solely infected with *Wolbachia*, solely infected with *Spiroplasma*, coinfected with both *Wolbachia* and *Spiroplasma*, and uninfected. Bioproject PRJNA475023 includes libraries exposed to abamectin and libraries not exposed to abamectin. PRJNA881040 includes samples from ordinary temperature and high temperature conditions, and Bioproject PRJNA636216 includes mites reared on distinct host plants. Host ribosomal protein S11 (rps), calmodulin-1 (cal), Histone H1 (H1), and Cyclooxygenase-2 (COX-2) genes were used as constitutive endogenous controls for comparison with virus abundance.

Following virome characterization, to assess virus prevalence across all analyzed samples, we cross-mapped each library to the 20 assembled putative viral sequences, including both endogenous and exogenous viruses (Figure 2). Except for AcPV, reads matching all viruses were present in at least two distinct samples, and most of them belonged to the same sequencing project. However, the dicistroviruses TtDV-1, TtDV-2, and AVT, together with the phenuiviruses GVT-1 and GVT-2, and the botourmiavirus TtOV were detected in libraries originated from independent projects (Figure 1). Of note, AVT and TtDV-1 were present in approximately 82% and 52% of the libraries, respectively (Figure 2). Interestingly, the dicistroviruses TtDV-1 and AVT, and the kitaviruses BVT-1 and BVT-2 showed normalized transcript abundance 10 to 40 times higher than constitutive genes (Figure 2). On the other hand, the Goukoviruses (GVT-1–4) showed the lowest abundance. Of note, EVE2 was the only endogenous viral element found in this work that showed to be transcriptionally active.

### *Wolbachia* presence impact on the viral dynamics

Upon evaluating the prevalence and distribution of viral contigs, a potential difference emerged between the virome composition of samples infected and uninfected by endosymbionts. This observation prompted an investigation into the potential impact of *Wolbachia* on the virome of *T. truncatus*, as seen in several other arthopods (Pimentel et al., 2021). To explore this hypothesis, we analyzed the levels of virus-derived transcripts across all treatments in the bioprojects PRJNA717652 and PRJNA644209. These datasets included samples infected exclusively with *Wolbachia* pipientis (W+S-) or *Spiroplasma* ixodetis (W-S+), as well as those under coinfection (W+S+) or uninfected (W-S-) conditions.

**FIGURE 3:**
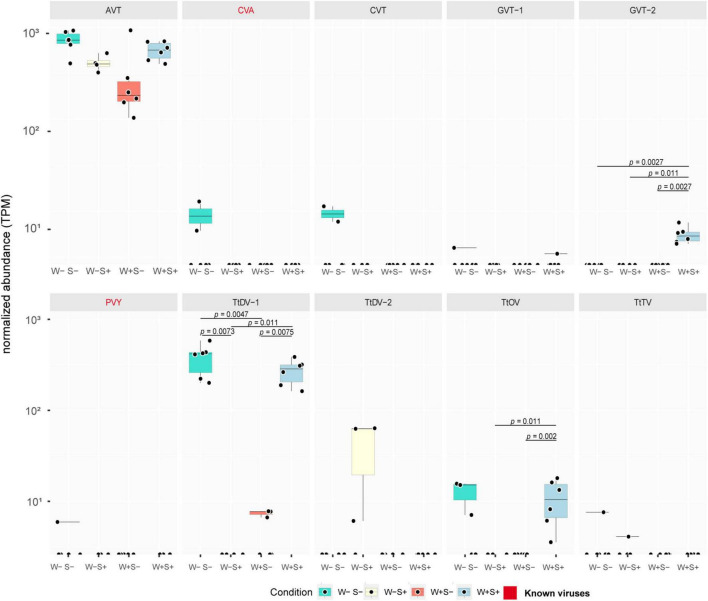
Abundance of virus-derived transcripts among different endosymbiont infection conditions. Boxplots illustrate TPM abundance of transcripts representing mite viruses in libraries subjected to distinct *Wolbachia* and *Spiroplasma* infection scenarios, including mites solely infected with *Wolbachia* (W+S-), *Spiroplasma* (W-S+), coinfected with both (W+S+), or uninfected (W-S-). Y-axis shows normalized TPM abundance while X-axis distinguishes between treatments. The statistical analysis was performed using Wilcoxon test and significance determined at a *p*-value < 0.05.

In the presence of *Wolbachia*, we observed a statistically significant decrease in the TPM levels of the polyadenylated (+)ssRNA TtDV-1 dicistrovirus. Compared to endosymbiont-uninfected mites, where the virus was consistently detected across all samples, the TPM levels dropped from nearly 10^3^ to less than 10^1^ TPM in half of the samples, while the virus was completely absent in the other half (Figure 3). For the other dicistrovirus, AVT, a slight decrease in TPM levels was observed, although it was not statistically significant. Interestingly, the non-polyadenylated botourmia virus TtOV, despite having lower TPM than TtDV-1, was detected in approximately half of the uninfected mite samples at levels around 10^1^ TPM, while it was completely absent in the W+S- samples (Figure 3).

**FIGURE 4:**
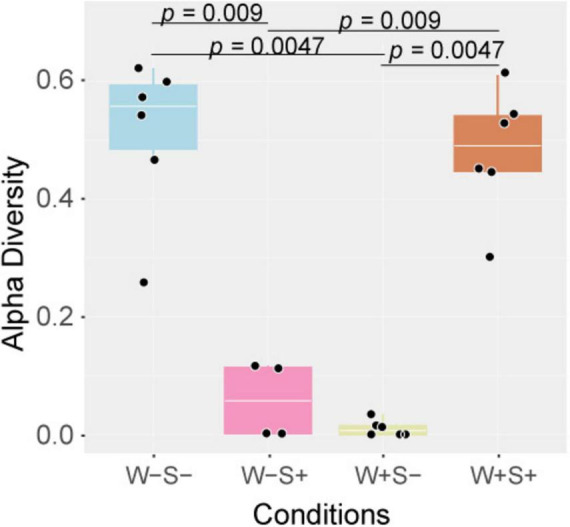
Alpha diversity is significantly altered by the presence of endosymbionts. Boxplot illustrating alpha diversity in libraries corresponding to different *Wolbachia* and *Spiroplasma* infection status, including mites infected with *Wolbachia* (W+S-) or *Spiroplasma* (W-S+) alone, coinfected with both (W + S +) or uninfected (W-S-). The Y-axis represents alpha diversity, and the X-axis categorizes libraries based on the sample conditions. The statistical analysis utilized the Wilcoxon test, with significance determined at a *p*-value < 0.05.

After evaluating the abundance of viral contigs, alpha diversity was assessed in the *Wolbachia*-infected samples. The W+S- samples displayed a notably lower alpha diversity in comparison to uninfected mites, suggesting that *Wolbachia* may interfere with the virome dynamics of *T. truncatus* (Figure 4). Consistent with this observation, uniform manifold approximation and projection (UMAP) analysis using the transcriptome and virome transcripts abundance of *T. truncatus*, revealed a clustering pattern where samples infected with *Wolbachia* segregated from co-infected and uninfected samples (Supplementary Figure 13).

### *Spiroplasma* shows a potential *Wolbachia*-like effect in impacting viral dynamics

Interestingly, when analyzing endosymbiont-related samples, the well-known viral-protective *Wolbachia* exhibited significant effects on viral dynamics. Separately, *Spiroplasma*, an endosymbiont associated with protection against nematodes and parasitoid wasps in some arthropods, also appeared to influence viral dynamics. Notably, in mites infected solely with *Spiroplasma*, the dicistrovirus TtDV-1 exhibited a drastic decrease in abundance, being completely absent from all samples when compared to the control, where it reached nearly 10^3^ TPM (Figure 3). This suggests that *Spiroplasma* may exert an even stronger impact than *Wolbachia* on the same viral transcript.

The impact on the abundance of the dicistrovirus AVT and the botourmiavirus TtOV was similar to that observed with *Wolbachia*. The AVT exhibited a slight decrease in abundance, while TtOV, which was present in approximately half of the libraries from uninfected mites, was completely absent in the *Spiroplasma* infection scenario. Notably, another dicistrovirus, TtDV-2, was detected exclusively in *Spiroplasma*-infected mites, the only population where TtDV-1 was absent (Figure 3).

Similar to the W+S- samples, mites infected solely with *Spiroplasma* exhibited lower viral alpha diversity compared to the control samples, although it was slightly higher than in mites infected solely with *Wolbachia* (Figure 4). This suggests that *Wolbachia* has a stronger impact on viral diversity than *Spiroplasma*. Supporting these findings, UMAP analysis showed that the whole transcriptome abundance of *Spiroplasma*-infected mites segregated from co-infected and uninfected samples but was closely clustered with the *Wolbachia*-infected mite population (Supplementary Figure 13).

### Co-infection possibly neutralize symbiont-driven virome suppression

In this distinct mite population, where both *Wolbachia* and *Spiroplasma* endosymbionts were present, despite both bacteria displaying potential effects on viral dynamics, a possible mutually antagonistic interaction was observed. The TtDV-1 virus, which showed decreased abundance in both W+S- and W-S+ populations, had its abundance restored to levels comparable to uninfected mites (nearly 10^3^ TPM) in the coinfection scenario (Figure 3). A similar effect was observed for the AVT virus, although the change was not statistically significant. Interestingly, TtOV, which was absent in solely infected samples and present in only half of the uninfected samples, was detected in all samples from the coinfection scenario (Figure 3). Additionally, the (-)ssRNA phenuivirus GVT-2 was exclusively detected in the coinfection samples and absent in all other conditions.

Interestingly, the coinfected samples exhibited high viral alpha diversity, comparable to that of the endosymbiont-uninfected mite population (Figure 4). In the UMAP analysis, the coinfected samples were distinctly segregated from the solely infected populations but clustered closely with the uninfected mite populations (Supplementary Figure 13). This suggests that the interaction between *Wolbachia* and *Spiroplasma* may influence viral dynamics in a unique way, potentially mitigating the observed individual impacts of each endosymbiont on the virome.

### Effect of temperature and abamectin treatment on the *T. truncatus* virome

Exploring the impact of abamectin exposure and different temperatures on virome composition and abundance of *T. truncatus*, we observed that *Potato virus Y* (PVY) and TtDV-2 were exclusively detected in *T. truncatus* samples collected from plants not exposed to abamectin (ABM–). Their abundance was approximately 10 and 100 TPM, respectively, suggesting a notable association between the absence of the pesticide and virus abundance. Conversely, TtNoV was exclusively identified in libraries containing *T. truncatus* exposed to abamectin (ABM+). Although not statistically significant, a distinct viral dynamic was observed in mites exposed to the pesticide (Supplementary Figure 14A).

On the other hand, temperature did not cause any significant change in virus abundance comparing ordinary and high temperature conditions (Supplementary Figure 14B). However, virus diversity analyses indicated that mites exposed to regular temperatures displayed lower alpha diversity compared to those subjected to high-temperature stress, although these differences were not statistically significant (Supplementary Figure 15A). Conversely, samples exposed to abamectin exhibited a more pronounced, although still not statistically significant, difference in alpha diversity compared to non-exposed individuals (Supplementary Figure 15B).

### Mite transcriptional responses to *Wolbachia* and/or *Spiroplasma* infection

**FIGURE 5:**
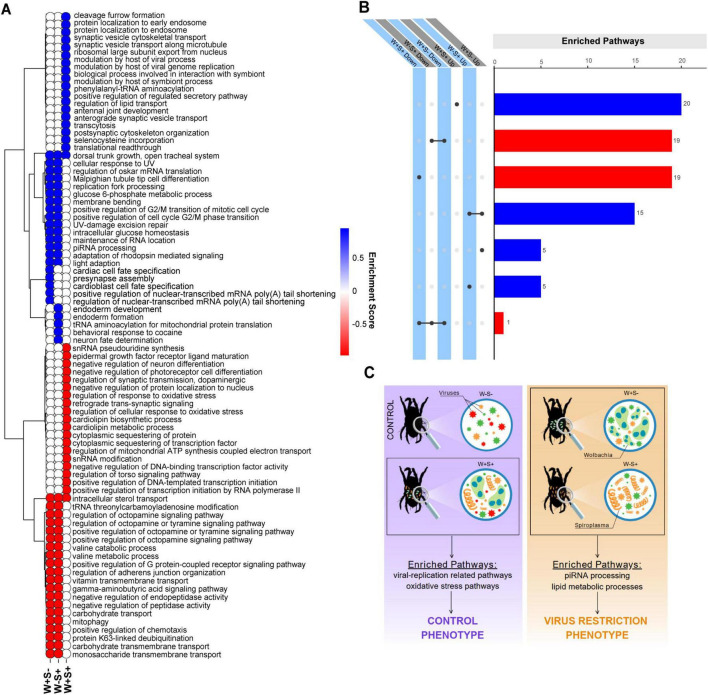
Transcriptionally altered pathways and gene sets during infection with *Wolbachia* and/or *Spiroplasma* in *T. truncatus* mites. Heatmap illustrating the top 20 enriched and bottom 20 enriched pathways identified through Gene Set Enrichment Analysis (GSEA) of *T. truncatus* naturally infected with *Wolbachia* alone (W+S-), *Spiroplasma* alone (W-S +), and *Wolbachia* and *Spiroplasma* coinfected individuals (W+S+). Up-regulated pathways are represented in blue while down-regulated pathways are shown in red **(A)**. The color gradient reflects the normalized enrichment score (NES) of each pathway. UpSet plot showing the intersections between the top 20 enriched and bottom 20 enriched pathways among W+ S-, W-S+ and W+S+ conditions. Up-regulated pathways are represented in blue while down-regulated pathways are shown in red **(B)**. Schematic representation of viral restriction phenotypes and mock phenotypes in various *T. truncatus* infection states. This schematic figure illustrates the viral restriction phenotypes observed in *T. truncatus* solely infected by *Wolbachia* (W+S-) and *Spiroplasma* (W-S+), as well as the control phenotypes observed in co-infected mites (W+S+) and uninfected mites (W-S-). The figure also highlights the enriched pathways associated with each infection state **(C)**.

Infection with *Wolbachia* and/or *Spiroplasma* significantly altered the virome composition and abundance of *T. truncatus*. Therefore, we investigated the transcriptional responses and pathways involved in the dynamics between endosymbiont bacteria and the host, focusing on the potential impact to virus infection. Gene Set Enrichment Analysis (GSEA) revealed several pathways enriched in during *Wolbachia* (W+S-) and *Spiroplasma* (W-S+) infection, many overlapping between conditions. Upregulated enriched pathways included piRNA processing, adaptation of rhodopsin-mediated signaling and membrane bending (Figures 5A, B, Supplementary Figures 16, 17 and Supplementary File 7). Notably, five unique pathways were significantly upregulated during *Wolbachia* or *Spiroplasma* infection alone. However, to our knowledge, none of these were yet described to have an association with antiviral activity (Figures 5A, B and Supplementary Figures 16, 17). Conversely, GSEA also identified significantly downregulated pathways that were shared between W+S- and W-S+ infected samples. These pathways included positive regulation of G protein-coupled receptor signaling, carbohydrate transport, octopamine regulation pathways and lipid metabolic processes. (Figures 5A, B, Supplementary Figures 16, 17 and Supplementary File 7).

Remarkably, GSEA analysis revealed a minimal overlap of enriched pathways between solely infected and co-infected samples with endosymbionts. The unique pathways enriched in upregulated genes in co-infected mites were associated with the host’s modulation of viral processes, including viral genome replication and interactions related to symbiosis. Additionally, pathways such as translation readthrough and the export of the ribosomal large subunit from the nucleus were also enriched. Conversely, pathways involved in the regulation of oxidative stress response, cardiolipin metabolism and biosynthesis, as well as the sequestering of proteins and transcription factors, were found to be downregulated. This differential regulation suggests a complex interplay between the host’s immune responses and viral dynamics in co-infected mites (Figures 5A, B and Supplementary Figure 18). A schematic illustration explaining the *Wolbachia*- and *Spiroplasma*-induced phenotypes can be visualized in the Figure 5C.

## Discussion

Metatranscriptomics is a powerful approach to understanding complex ecosystems and has illuminated the interplay between microorganisms and their hosts (Bashiardes et al., 2016; François et al., 2019). Our metatranscriptomic analysis of the important agricultural pest *T. truncatus* using data obtained from 39 libraries publicly available offered a glimpse into its virome landscape. Overall, most of the viral families identified have elements known to infect mites such as *Dicistroviridae* (Niu et al., 2019) and *Nodaviridae* (François et al., 2019). Furthermore, we have also identified viruses from the families *Phenuiviridae* (Yadav et al., 2019) and *Nudiviridae* (Harrison et al., 2020), both known to infect arthropods. In addition, we have identified previously known and several new viruses associated with families that are traditionally associated with plants including *Kitaviridae*, *Botourmiaviridae*, *Virgaviridae*, *Betaflexiviridae*, and *Potyviridae*. Of note, transmission by mites of viruses belonging to these families has been described, as exemplified by the transmission of viruses from the *Kitaviridae* family by Ramos-González et al. (2022), Tassi et al. (2022), while virgaviruses transmission has been described by Pleshakova et al. (2018). Furthermore, the mite-mediated transmission of *Betaflexiviridae* has been described by Bertazzon et al. (2021) and *Potyviridae* transmission by Choi et al. (1999). We could speculate that viruses identified in our study can potentially be transmitted to plants by *T. truncatus*. This hypothesis was supported by the identification of *Potato virus Y* and *Cherry virus A*, both known to infect economically important crops (Schulz, 1963; Karasev and Gray, 2013; Simkovich et al., 2021). The viruses identified in this study were classified into viral families including, the *Dicistroviridae* and *Nudiviridae* families, both of which are known to hold significant potential for arthropod control strategies (Bonning and Miller, 2010). Dicistroviruses, including *Cricket paralysis virus* (CrPV) (Manousis and Moore, 1987), *Rhopalosiphum padi virus* (RhPV) (D’Arcy et al., 1981), and *Homalodisca coagulata* virus *1* (HoCV-1) (Biesbrock et al., 2014), have been demonstrated to negatively impact arthropod populations. Similarly, Nudiviruses, which infect various arthropods, can impair development and reduce fertility in pest species (Prasad and Srivastava, 2016). Both viral families offer environmentally friendly alternatives to chemical pesticides, although further research is essential to validate the finds and fully understand the potential applications of these characterized viruses in broader pest control strategies.

Our study revealed a substantial impact in the abundance of specific viral sequences in *T. truncatus* when infected alone with either *Wolbachia* or *Spiroplasma*. Notably, the dicistrovirus TtDV-1, a virus in our study, is the most impacted by endosymbiont’s influence, with a considerable impact on virus abundance. Nevertheless, a distinct scenario emerges when considering populations co-infected with both *Wolbachia* and *Spiroplasma* (W+S+) or those uninfected (W-S-). Our analyses pointed out to a reduction in virus abundance of (+)ssRNA viruses in *Wolbachia*-infected samples, which is aligned with the *Wolbachi’s* established role in regulating viral replication in other species (Moreira et al., 2009; Mousson et al., 2010; Hoffmann et al., 2011; Aliota et al., 2016; Caragata et al., 2016; Edenborough et al., 2021; Loterio et al., 2024). A similar effect has also been reported in the dicistrovirus *Cricket paralysis virus* and *Drosophila C virus* (DCV) in *D. melanogaster* (Bonning, 2009). Moreover, we were not able to detect TtDV-1 in *Spiroplasma* infected samples, indicating a potentially stronger inhibition of virus abundance in comparison to *Wolbachia*. Both TtOV and AVT exhibited comparable outcomes, revealing a consistent pattern of diminished viral abundance. It is important to point out that the virus restriction effect was evident across both polyadenylated viruses (TtDV-1 and AVT) and the non-polyadenylated botourmia virus TtOV, suggesting it is an authentic mechanism instead of an artifact due to the strategy used to build the sequencing libraries.

When analyzing viral abundance in singly infected mites, we saw that *Spiroplasma* suppressed the dicistrovirus TtDV-1, permitting TtDV-2 to predominate, a pattern reminiscent of competitive release between distinct arboviral genotypes (Norton et al., 2020). In *Aedes* and other arthropods, *Wolbachia* similarly diminishes viral diversity and load (Sinkins, 2004; Moreira et al., 2009; Hoffmann et al., 2011; De Oliveira et al., 2015; Ant et al., 2020). Of note, work in *T. truncatus* confirms that co-infecting symbionts engage in hierarchical competition: *Wolbachia* typically outcompetes *Spiroplasma* in double infections, achieving higher densities and inducing stronger cytoplasmic incompatibility than *Spiroplasma* alone (Yang et al., 2021). In addition, Xie et al. (2020) showed that co-infection can impose fitness costs yet restore CI phenotypes lost in single infections, underscoring the antagonistic interplay between these bacteria.

Mechanistically, both *Wolbachia* and *Spiroplasma* can induce CI—although sometimes weak—in *T. truncatus*, disrupting host reproduction and limiting vertical viral transmission (Xie et al., 2020). In other species it has been shown that they also compete for intracellular resources—space, nutrients, and host molecular machinery—thereby constraining viral replication niches (Werren et al., 2008; Sinkins, 2013; Jupatanakul et al., 2014; Hrdina et al., 2024). *Spiroplasma* uniquely produces ribosome-inactivating proteins that defend against nematodes and parasitoids (Ballinger and Perlman, 2019; Jones and Hurst, 2020) and may similarly inhibit viral translation in mites. Additionally, endosymbionts can modulate the host’s immune system by enhancing RNA interference pathways and altering gene expression, thereby strengthening antiviral defenses (Ou et al., 2022; Mushtaq et al., 2025).

In this study, mites infected with either *Wolbachia* or *Spiroplasma* alone exhibited transcriptional regulation targeting genes involved in several pathways potentially linked to *Wolbachia*’s antiviral mechanisms, as described by Mushtaq et al., 2025. Notably, one of the most prominent enriched pathways among upregulated genes was the piRNA processing pathway. This finding is particularly noteworthy as *Wolbachia* has been described to modulate host piRNAs (Hess et al., 2011; Aguiar et al., 2016; Etebari et al., 2016; Hussain et al., 2016). Overall, RNAi pathways including the small interfering RNAs (siRNAs) are well described to control arbovirus infections in mosquitoes and other arthropods, and recent research are providing evidence that the piRNA pathway might also contribute to antiviral activity (Wu et al., 2010; Blair, 2011; Donald et al., 2012; Morazzani et al., 2012; Schnettler et al., 2013). These results provide evidence that multifactorial events resulting of *Wolbachia* or *Spiroplasma* infections could explain the changes in viral abundance and diversity observed. Supporting this hypothesis, we also observed that autophagy was enriched by both endosymbionts’ infection. This important pathway is involved in cellular degradation previously shown to be activated by *Wolbachia* and *Spiroplasma* infection and can function as an antiviral host response (Tallóczy et al., 2006; Voronin et al., 2012; Sinkins, 2013; Jheng et al., 2014; Ou et al., 2022).

Analysis of downregulated gene sets and pathways in *Wolbachia-* and *Spiroplasma*-exclusively infected mites revealed a link to lipid metabolism, particularly sterol and steroid metabolic processes. Cholesterol, the end-product of this pathway, is a vital molecule targeted by various viruses, including Dengue virus. Modulation of cholesterol dynamics and metabolism is a strategy employed by the host’s innate immunity to combat viral infections (Haas and Mooradian, 2010; Blanc et al., 2011). Interestingly, *Wolbachia* lacks the ability to synthesize cholesterol itself (Wu et al., 2004; Molloy et al., 2016). This dependency leads to competition with the host for lipid molecules, a mechanism proposed to contribute to *Wolbachia*-mediated viral blocking (Caragata et al., 2013; Koh et al., 2020). *Wolbachia* and *Spiroplasma* are known to deplete lipid availability, creating an unfavorable environment for viral replication (Koh et al., 2020) and larval development (Paredes et al., 2016). Furthermore, lipid metabolic processes are linked to changes in the membrane lipid composition of host cells, which can be critical for the formation of replication complexes for (+)ssRNA viruses (Loterio et al., 2024). Further research is needed to explore the potential antiviral capabilities of *Wolbachia* and *Spiroplasma* and the specific mechanisms involved in providing protection against viral infections in *T. truncatus* (Hamilton et al., 2016; Ballinger and Perlman, 2019).

Notably, W+S+ populations exhibited similar diversity and abundance patterns to those of W-S- populations, suggesting that the putative virus-blocking effect of endosymbionts may be compromised in co-infection scenarios. Interestingly, the co-infected mite population displayed minimal overlap in enriched pathways compared to mites solely infected with *Wolbachia* or *Spiroplasma*. This distinction highlights the unique enrichment of host defense-specific pathways in the co-infected group. These enriched pathways encompass processes that modulate viral processes, such as viral genome replication, alongside pathways involved in symbiosis-related interactions. This implies that, despite the presence of endosymbionts, the host is actively responding to viral infections. While oxidative stress is a typical host response to viral infection, it appears to be connected to *Wolbachia*’s presence (Wong et al., 2015). Studies have shown a link between *Wolbachia*’s antiviral function and increased oxidative stress levels (Wong et al., 2015; Zug and Hammerstein, 2015). Interestingly, *Spiroplasma* acts in opposition. It promotes the production of antioxidants (Ding, 2022) which could potentially counteract the oxidative stress and weaken *Wolbachia*’s antiviral effects in coinfected samples, where oxidative stress pathway is found enriched in downregulated genes.

Interestingly, several pathways associated with viral replication were found to be enriched. These include translation readthrough, protein and transcription factor sequestration, and the export of the ribosomal large subunit from the nucleus. Viruses have evolved sophisticated mechanisms to hijack host cellular machinery for their replication (Jaafar and Kieft, 2019). During infection, viruses exploit host ribosomes to translate viral mRNA into proteins essential for viral replication and assembly (Li, 2019). Additionally, viruses sequester host transcription factors and proteins, redirecting them to facilitate viral gene expression while suppressing host defenses (Ahlquist et al., 2003; Den Boon et al., 2010). This sequestration often involves the manipulation of RNA-binding proteins, which play crucial roles in RNA metabolism and gene regulation (Serge Andigema, 2024). Furthermore, some viruses employ translation readthrough strategies, allowing ribosomes to bypass stop codons and produce extended viral proteins that enhance viral replication and pathogenicity (Cimino et al., 2011; Prasad et al., 2024). Such findings raise questions about the interplay between endosymbionts and host immune responses, which reinforces the need for further investigation into the mechanisms that are basis for these interactions. A deeper understanding of the relationship between symbionts is particularly crucial, especially considering the potential release of Aedes mosquitoes infected with *Wolbachia* strains exhibiting imperfect infection blocking. This could exert selective pressure on viral populations (Salje and Jiggins, 2024), and if our findings are further confirmed, co-infections with *Spiroplasma* may undermine *Wolbachia*’s antiviral efficacy, potentially compromising research efforts aimed at reducing arbovirus transmission (Hoffmann et al., 2011; Murray et al., 2016; Salje and Jiggins, 2024).

Environmental factors, such as temperature and pesticide exposure, play significant roles in shaping mite viromes and viral dynamics (Mourier and Poulsen, 2000; Bounfour and Tanigoshi, 2001; Varghese et al., 2016; Li et al., 2023). In our study, *T. truncatus* specimens under heat-stressed conditions exhibited higher viral diversity, although no statistically significant differences were observed in diversity or viral abundance. Controversially, abamectin exposure influenced virome composition, with PVY and TtDV-2 exclusively detected in non-exposed mites, suggesting a potential suppressive effect of the pesticide. This aligns with studies showing that abamectin can inhibit viral RNA and protein synthesis in alphaviruses like Chikungunya virus (Varghese et al., 2016). Conversely, TtNoV was predominantly found in abamectin-exposed mites, indicating a selective impact of the pesticide on virome structure. These effects may stem from both temperature and abamectin altering mite fitness, thereby influencing their susceptibility to viral infections. While temperature stress modulates virome diversity without statistical significance, abamectin may exert antiviral properties or reshape virome composition through other mechanisms. Further investigation is required to elucidate these complex interactions and their implications for pest control and agricultural ecosystems.

This study provides a comprehensive analysis of the virome of *T. truncatus*, a significant agricultural pest. Through metatranscriptomics, we identified both known and novel viral species, underscoring *T. truncatus*’ potential as an emerging vector for important plant viruses, such *as Potato Virus Y* and *Cherry Virus A*. Our results indicate that abiotic factors, particularly abamectin, diminish viral abundance, suggesting a complex interplay between pesticide use and viral dynamics. Moreover, our investigation into the effects of endosymbionts reveals that single infections with *Wolbachia* or *Spiroplasma* led to a notable decrease in both viral abundance and diversity, particularly dicistroviruses. However, co-infection with these symbionts appears to negate this antiviral effect, suggesting that their interactions may result in competitive dynamics between endosymbionts, further influencing viral replication and transmission. It is important to note that these findings were drawn from publicly available RNA-Seq datasets, with control and treatment conditions carefully compared within libraries from the same study to minimize potential biases from genetic background variations. Further experimental validation is necessary to confirm the observed effects of abiotic factors and endosymbionts on the virome of the spider mite. Overall, our findings contribute to shed light on the intricate relationships between the virome of *T. truncatus*, its microbial associates, and abiotic factors, highlighting the need for further research into these interactions to inform pest management strategies in agricultural systems. Understanding these dynamics will be crucial for developing effective control measures against viral diseases in crops.

## Data Availability

The scripts used to perform differential expression and diversity analysis are available at https://github.com/LymF/Truncatus-paper. The accession codes of public data analyzed in our work are provided in the Supplementary File 1. The viral sequences assembled in this work were deposited at NCBI Third Party Annotation database under the accession numbers (TPA: BK066973-BK066986, TPA: BK066963-BK066972, and TPA: BK067800-BK067802).
