# Supplementary material for: Potential effect of Wolbachia on virus restriction in the spider mite T. truncatus
Source: Front Microbiol. 2025 May 29;16:1570606. doi: 10.3389/fmicb.2025.1570606 (PMC12159000; doi:10.3389/fmicb.2025.1570606)
Supplement: Supplementary file 1 [file Data_Sheet_1.zip › Figure S12.pdf]

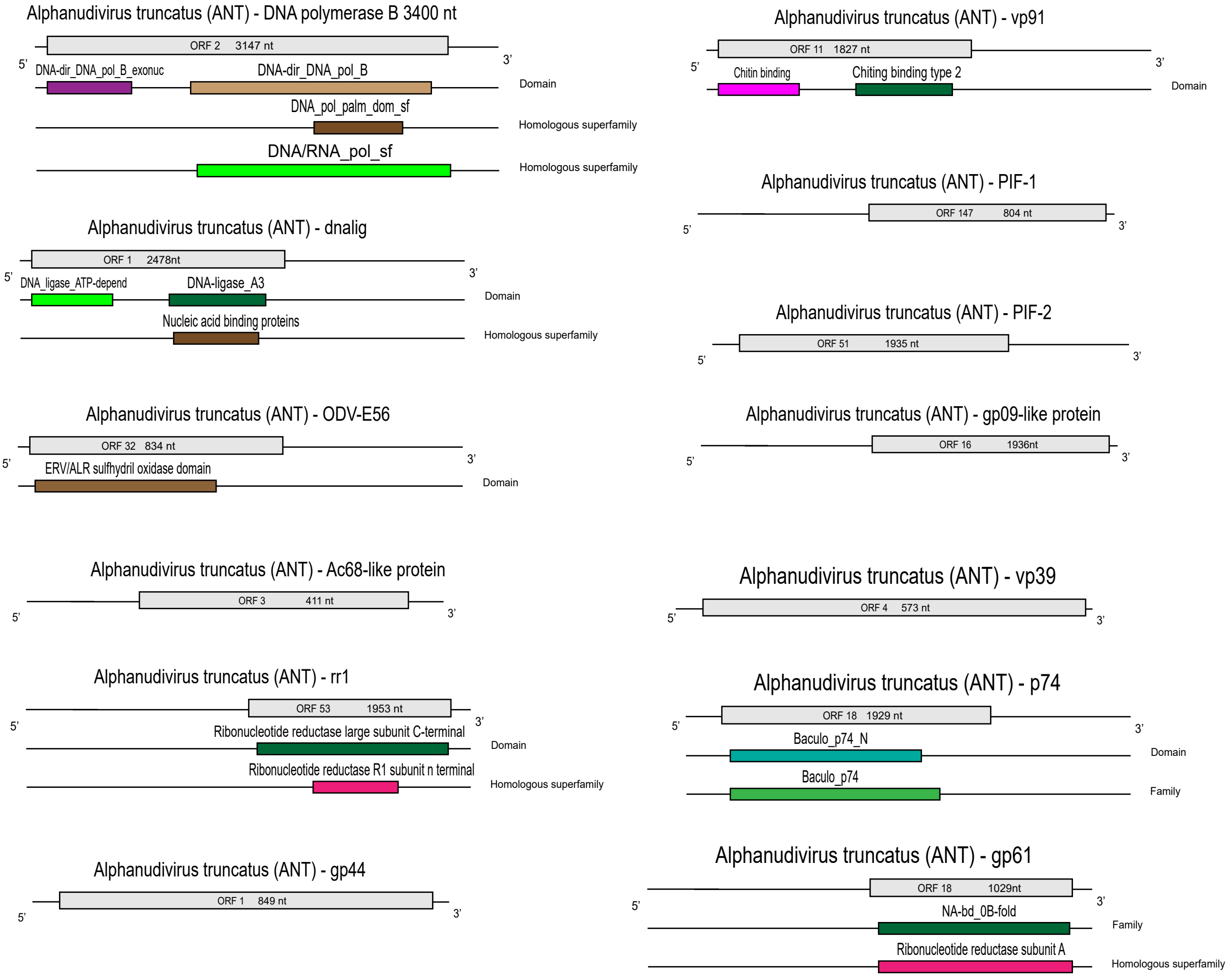
Alphanudivirus truncatus (ANT) - DNA polymerase B 3400 nt
ORF 2 3147 nt
5'
3'
DNA-dir_DNA_pol_B_exonuc
DNA-dir_DNA_pol_B
Domain
DNA_pol_palm_dom_sf
Homologous superfamily
DNA/RNA_pol_sf
Homologous superfamily
Alphanudivirus truncatus (ANT) - dnalig
ORF 1 2478nt
5'
3'
DNA_ligase_ATP-depend
DNA-ligase_A3
Domain
Nucleic acid binding proteins
Homologous superfamily
Alphanudivirus truncatus (ANT) - ODV-E56
ORF 32 834 nt
5'
3'
ERV/ALR sulfhydril oxidase domain
Domain
Alphanudivirus truncatus (ANT) - Ac68-like protein
ORF 3 411 nt
5'
3'
Alphanudivirus truncatus (ANT) - rr1
ORF 53 1953 nt
5'
3'
Ribonucleotide reductase large subunit C-terminal
Domain
Ribonucleotide reductase R1 subunit n terminal
Homologous superfamily
Alphanudivirus truncatus (ANT) - gp44
ORF 1 849 nt
5'
3'
Alphanudivirus truncatus (ANT) - vp91
ORF 11 1827 nt
5'
3'
Chitin binding
Chiting binding type 2
Domain
Alphanudivirus truncatus (ANT) - PIF-1
ORF 147 804 nt
5'
3'
Alphanudivirus truncatus (ANT) - PIF-2
ORF 51 1935 nt
5'
3'
Alphanudivirus truncatus (ANT) - gp09-like protein
ORF 16 1936nt
5'
3'
Alphanudivirus truncatus (ANT) - vp39
ORF 4 573 nt
5'
3'
Alphanudivirus truncatus (ANT) - p74
ORF 18 1929 nt
5'
3'
Baculo_p74_N
Domain
Baculo_p74
Family
Alphanudivirus truncatus (ANT) - gp61
ORF 18 1029nt
5'
3'
NA-bd_OB-fold
Family
Ribonucleotide reductase subunit A
Homologous superfamily
