# Supplementary material for: Potential effect of Wolbachia on virus restriction in the spider mite T. truncatus
Source: Front Microbiol. 2025 May 29;16:1570606. doi: 10.3389/fmicb.2025.1570606 (PMC12159000; doi:10.3389/fmicb.2025.1570606)
Supplement: Supplementary file 1 [file Data_Sheet_1.zip › Figure S9.pdf]

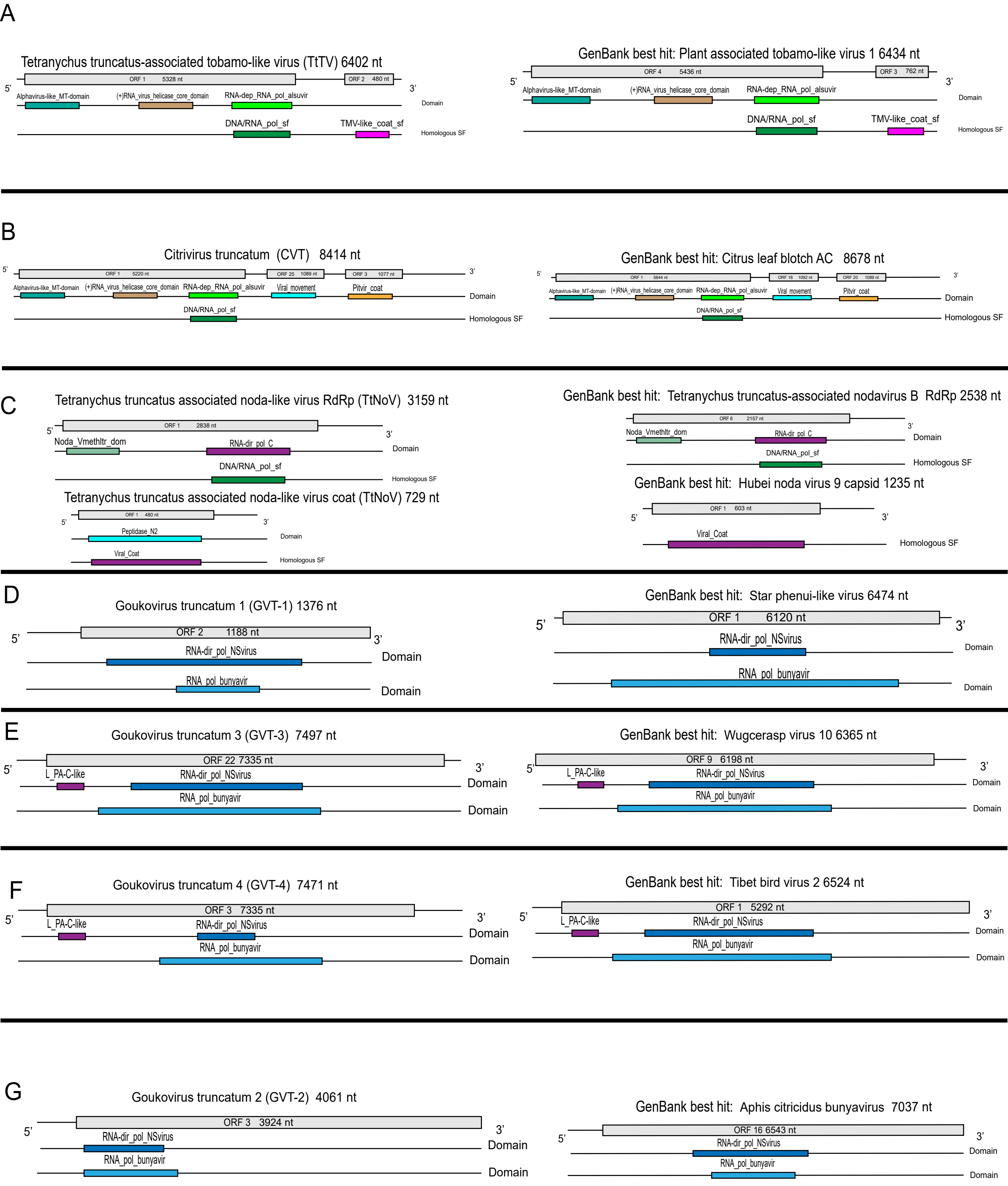
A
Tetranychus truncatus-associated tobamo-like virus (TtTV) 6402 nt
5'
ORF 1 5328 nt
ORF 2 480 nt
3'
Alphavirus-like_MT-domain
(+)RNA_virus_helicase_core_domain
RNA-dep_RNA_pol_alsuvir
Domain
DNA/RNA_pol_sf
TMV-like_coat_sf
Homologous SF
GenBank best hit: Plant associated tobamo-like virus 1 6434 nt
ORF 4 5436 nt
ORF 3 762 nt
Alphavirus-like_MT-domain
(+)RNA_virus_helicase_core_domain
RNA-dep_RNA_pol_alsuvir
DNA/RNA_pol_sf
TMV-like_coat_sf
B
Citrivirus truncatum (CVT) 8414 nt
ORF 1 5220 nt
ORF 25 1089 nt
ORF 3 1077 nt
Alphavirus-like_MT-domain
(+)RNA_virus_helicase_core_domain
RNA-dep_RNA_pol_alsuvir
Viral_movement
Pitvir_coat
DNA/RNA_pol_sf
GenBank best hit: Citrus leaf blotch AC 8678 nt
ORF 1 5844 nt
ORF 18 1092 nt
ORF 20 1089 nt
C
Tetranychus truncatus associated noda-like virus RdRp (TtNoV) 3159 nt
ORF 1 2838 nt
Noda_Vmethltr_dom
RNA-dir_pol_C
DNA/RNA_pol_sf
Tetranychus truncatus associated noda-like virus coat (TtNoV) 729 nt
ORF 1 480 nt
Peptidase_N2
Viral_Coat
GenBank best hit: Tetranychus truncatus-associated nodavirus B RdRp 2538 nt
ORF 6 2157 nt
GenBank best hit: Hubei noda virus 9 capsid 1235 nt
ORF 1 603 nt
D
Goukovirus truncatum 1 (GVT-1) 1376 nt
ORF 2 1188 nt
RNA-dir_pol_NSvirus
RNA_pol_bunyavir
GenBank best hit: Star phenui-like virus 6474 nt
ORF 1 6120 nt
E
Goukovirus truncatum 3 (GVT-3) 7497 nt
ORF 22 7335 nt
L_PA-C-like
GenBank best hit: Wugcerasp virus 10 6365 nt
ORF 9 6198 nt
F
Goukovirus truncatum 4 (GVT-4) 7471 nt
ORF 3 7335 nt
GenBank best hit: Tibet bird virus 2 6524 nt
ORF 1 5292 nt
G
Goukovirus truncatum 2 (GVT-2) 4061 nt
ORF 3 3924 nt
GenBank best hit: Aphis citricidus bunyavirus 7037 nt
ORF 16 6543 nt
