# Supplementary figures and images for: Potential effect of Wolbachia on virus restriction in the spider mite T. truncatus
Source: Front Microbiol. 2025 May 29;16:1570606. doi: 10.3389/fmicb.2025.1570606 (PMC12159000; doi:10.3389/fmicb.2025.1570606)

**A**

## Potato Virus Y (PVY) 9595 nt

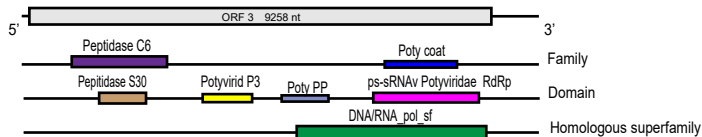**B**

## Cherry Virus A (CVA) 6397 nt

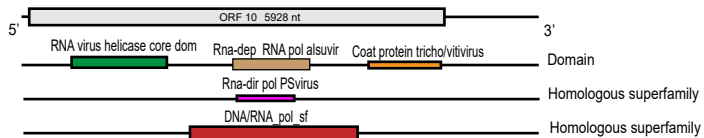**C**

## Acyrtosiphon pisum virus (AcPV) 10054 nt

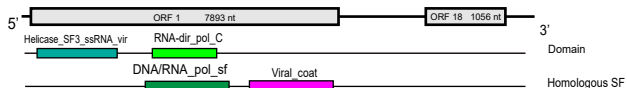

Supplement: Supplementary file 1 [file Data_Sheet_1.zip › Figure S1.pdf]

Tree scale: 1

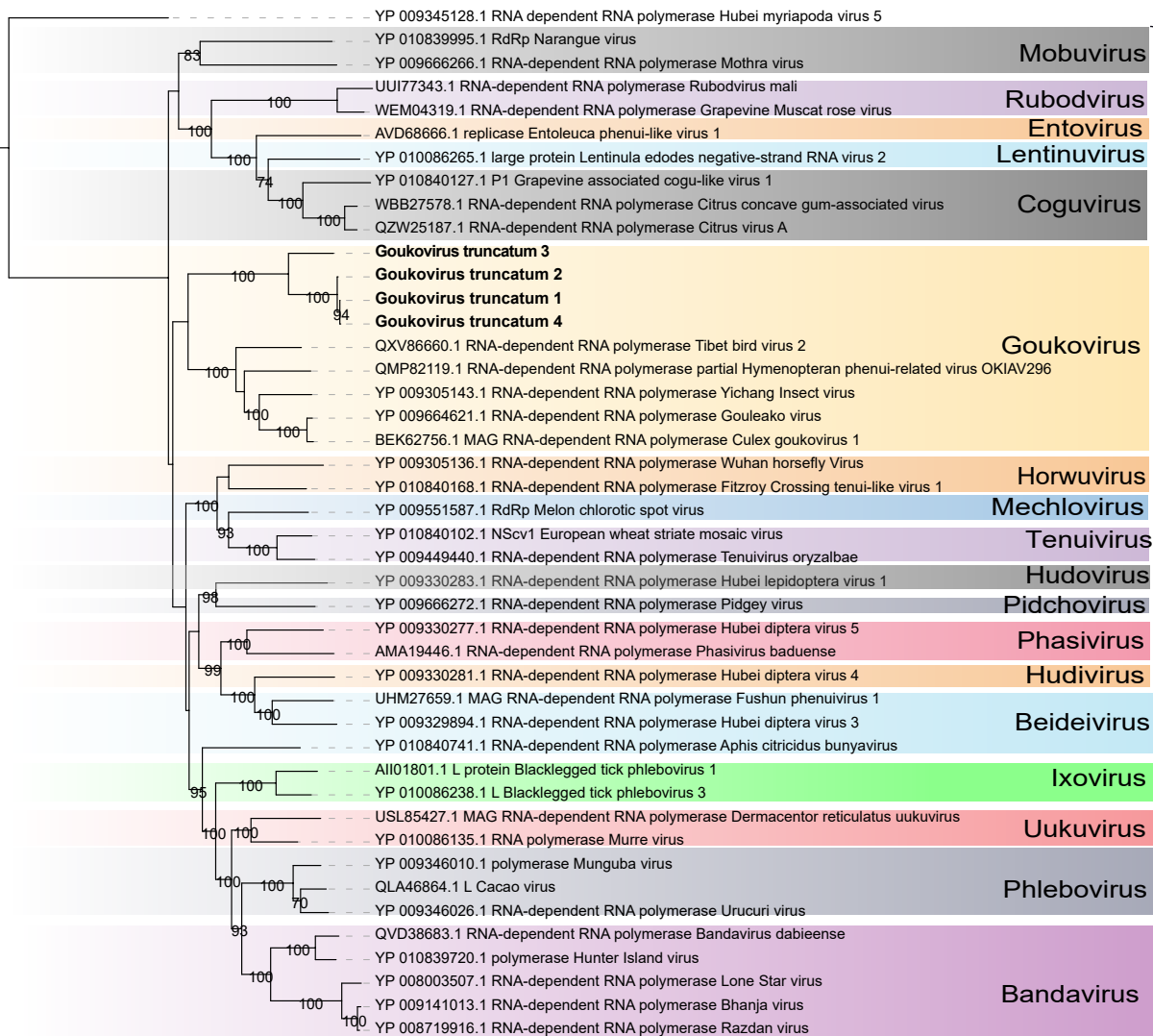

Phenuiviridae

Supplement: Supplementary file 1 [file Data_Sheet_1.zip › Figure S10.pdf]

Tree scale: 1

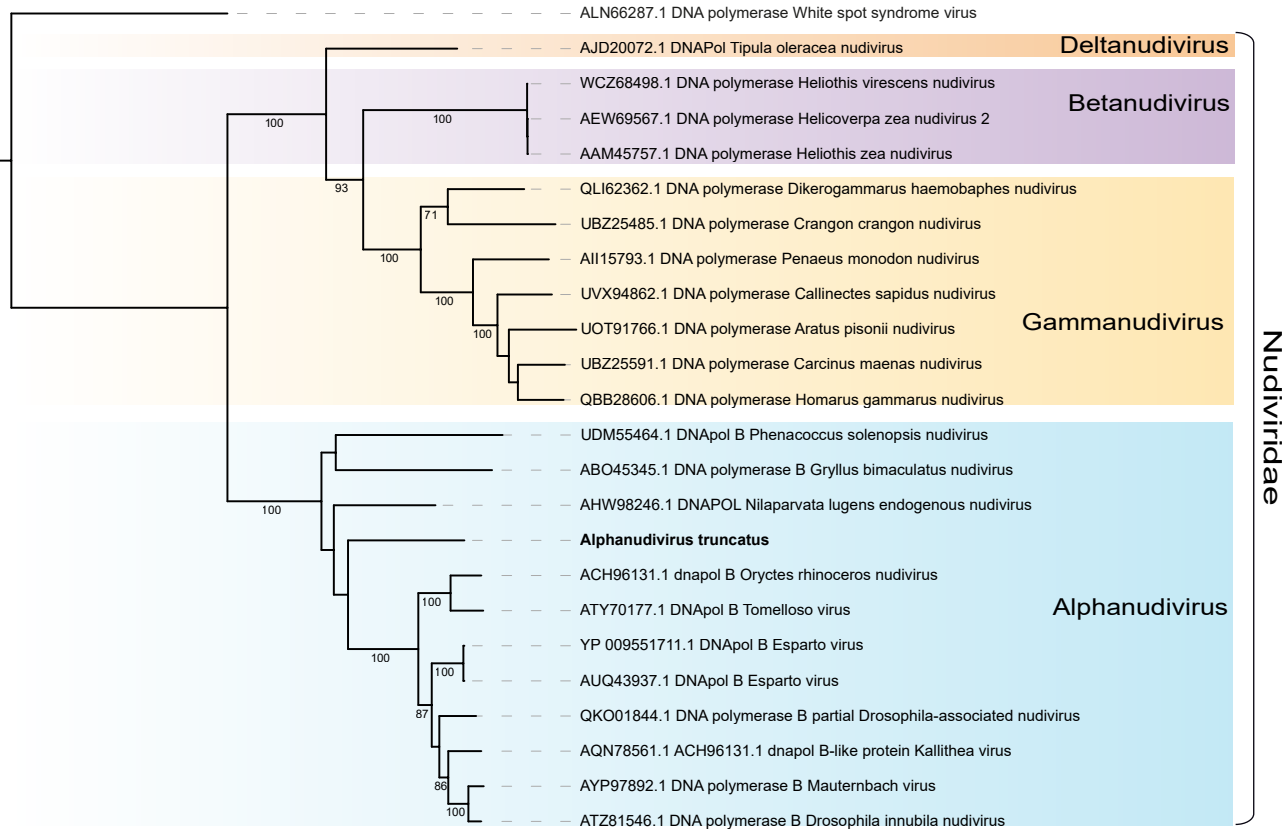

Supplement: Supplementary file 1 [file Data_Sheet_1.zip › Figure S11.pdf]

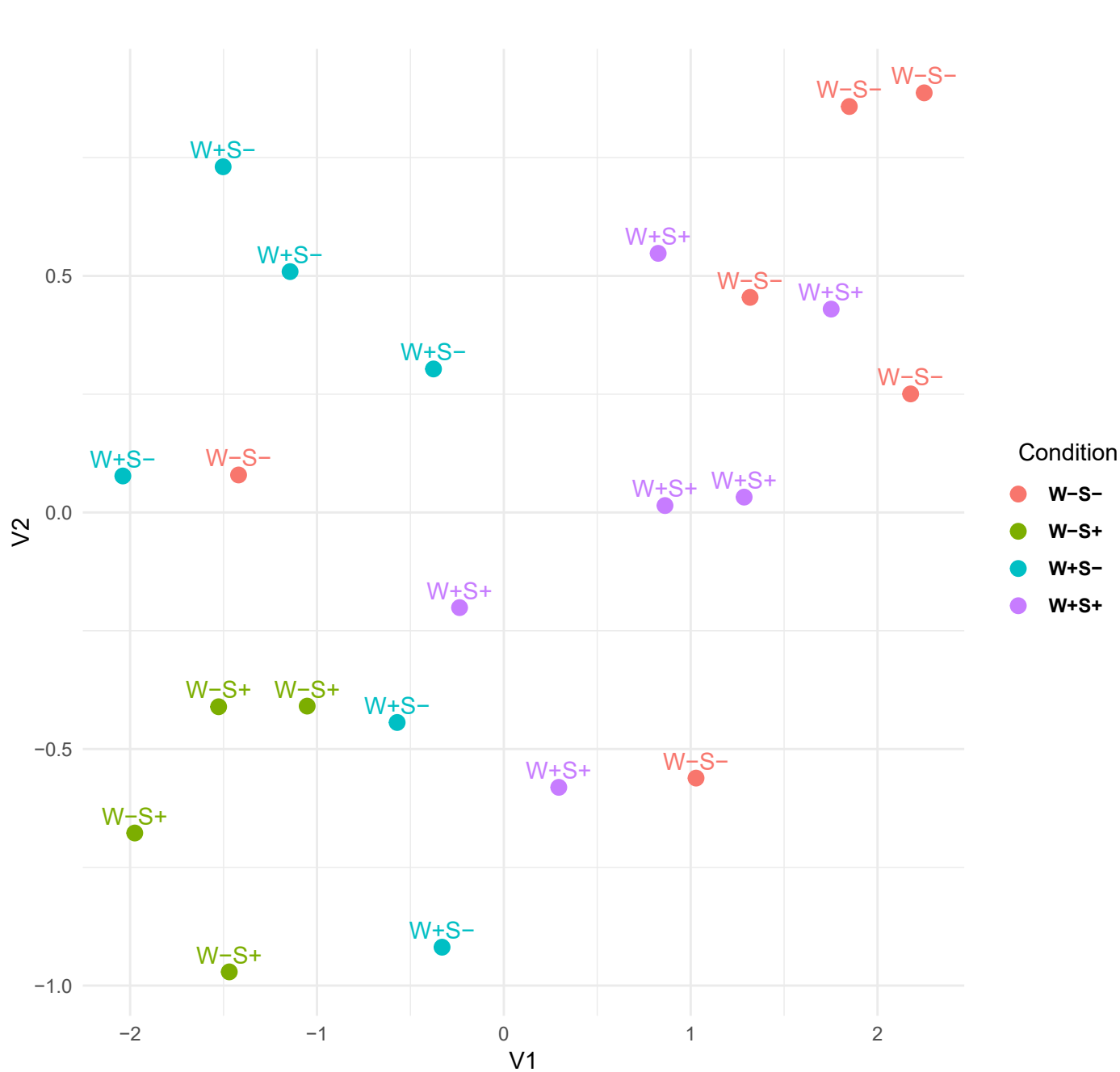

Supplement: Supplementary file 1 [file Data_Sheet_1.zip › Figure S13.pdf]

**A**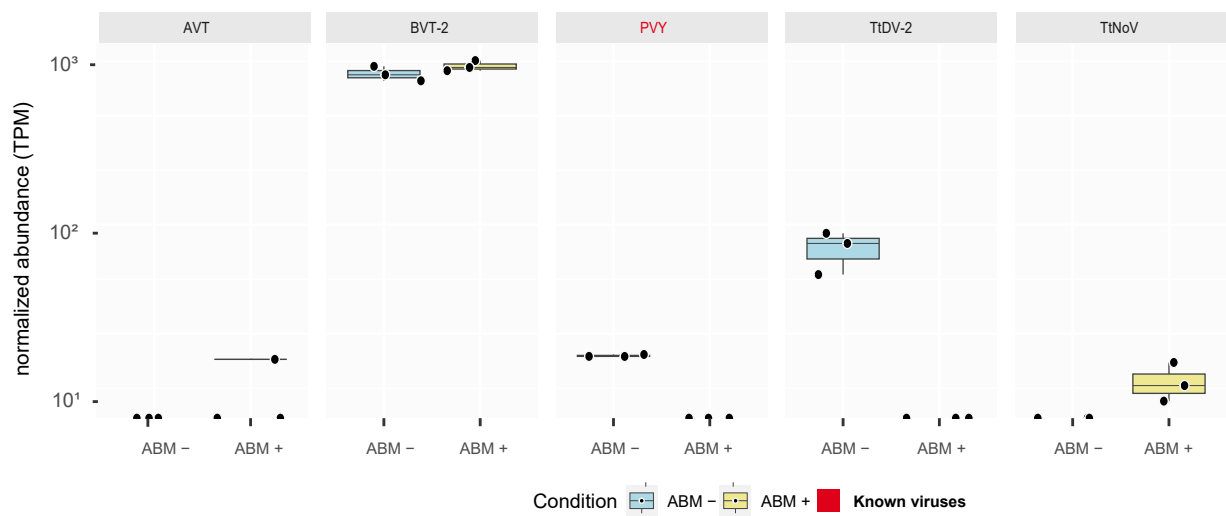**B**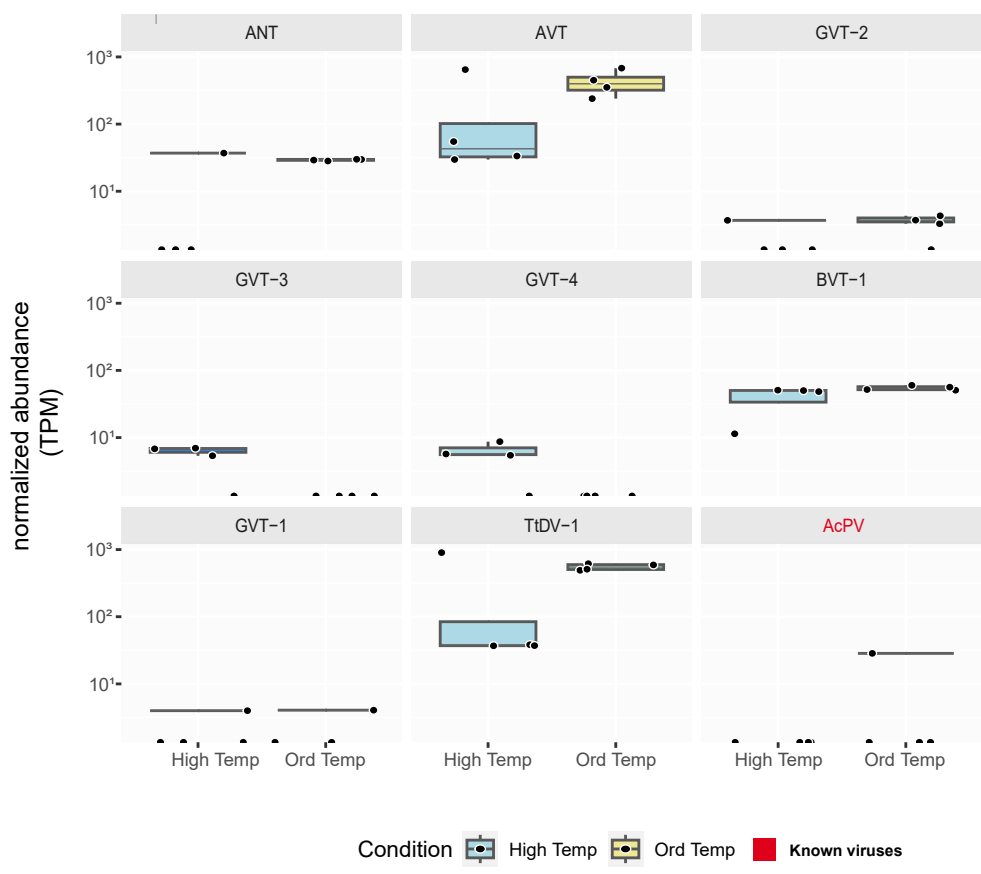

Supplement: Supplementary file 1 [file Data_Sheet_1.zip › Figure S14.pdf]

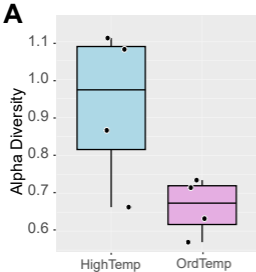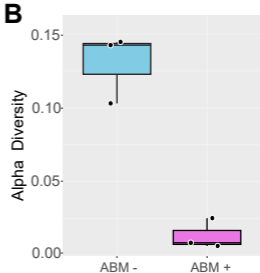

Supplement: Supplementary file 1 [file Data_Sheet_1.zip › Figure S15.pdf]

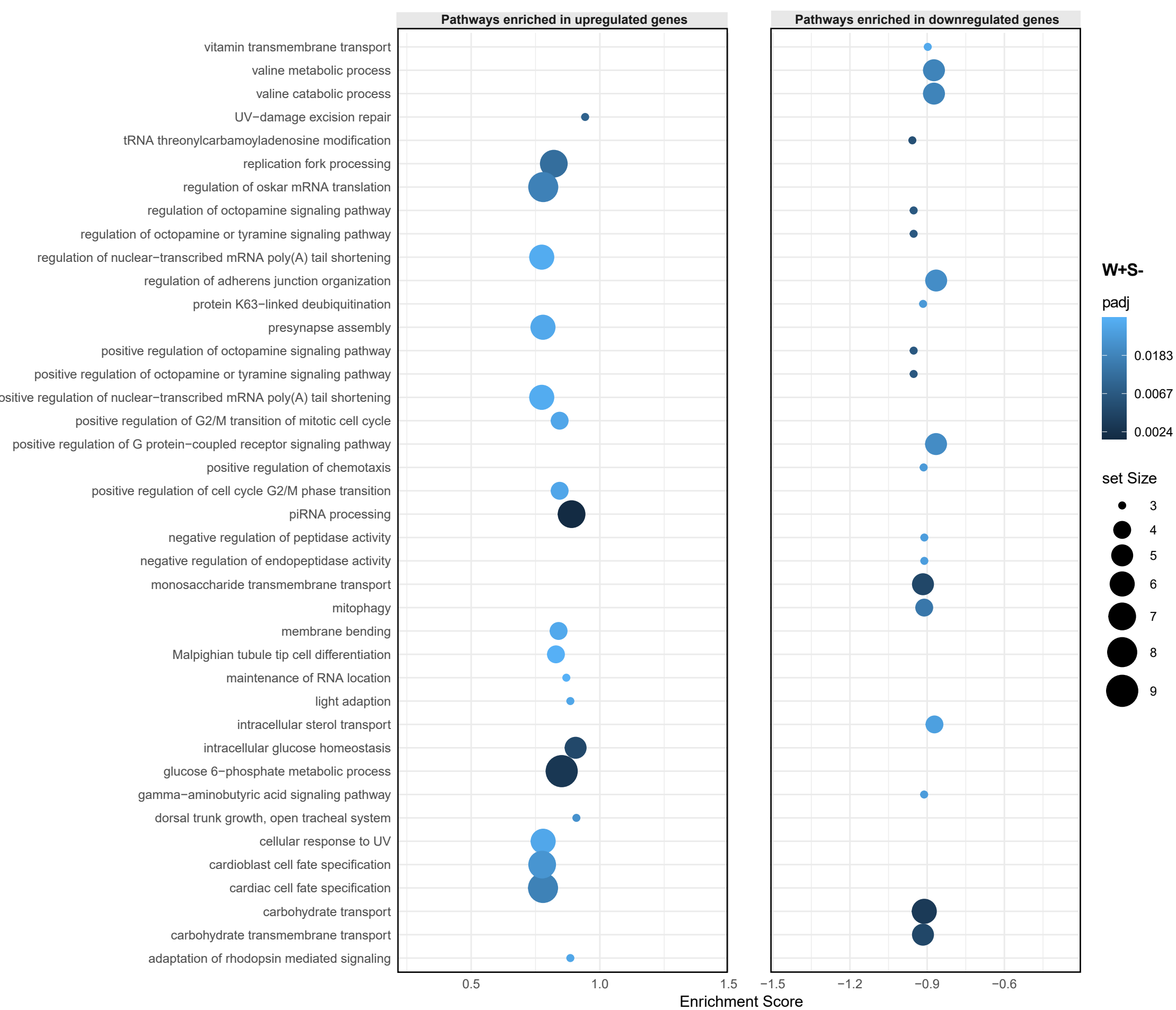

Supplement: Supplementary file 1 [file Data_Sheet_1.zip › Figure S16.pdf]

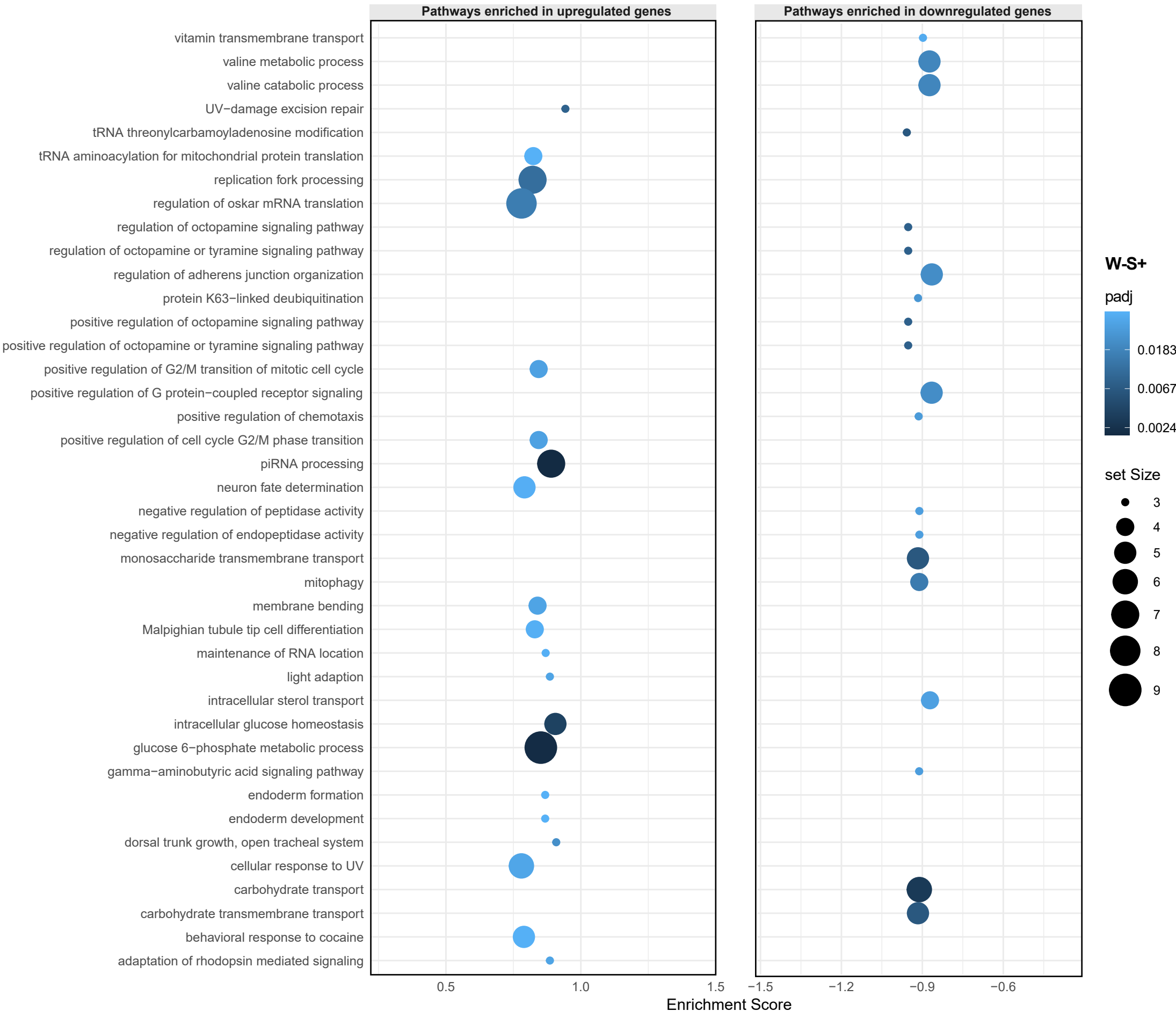

Supplement: Supplementary file 1 [file Data_Sheet_1.zip › Figure S17.pdf]

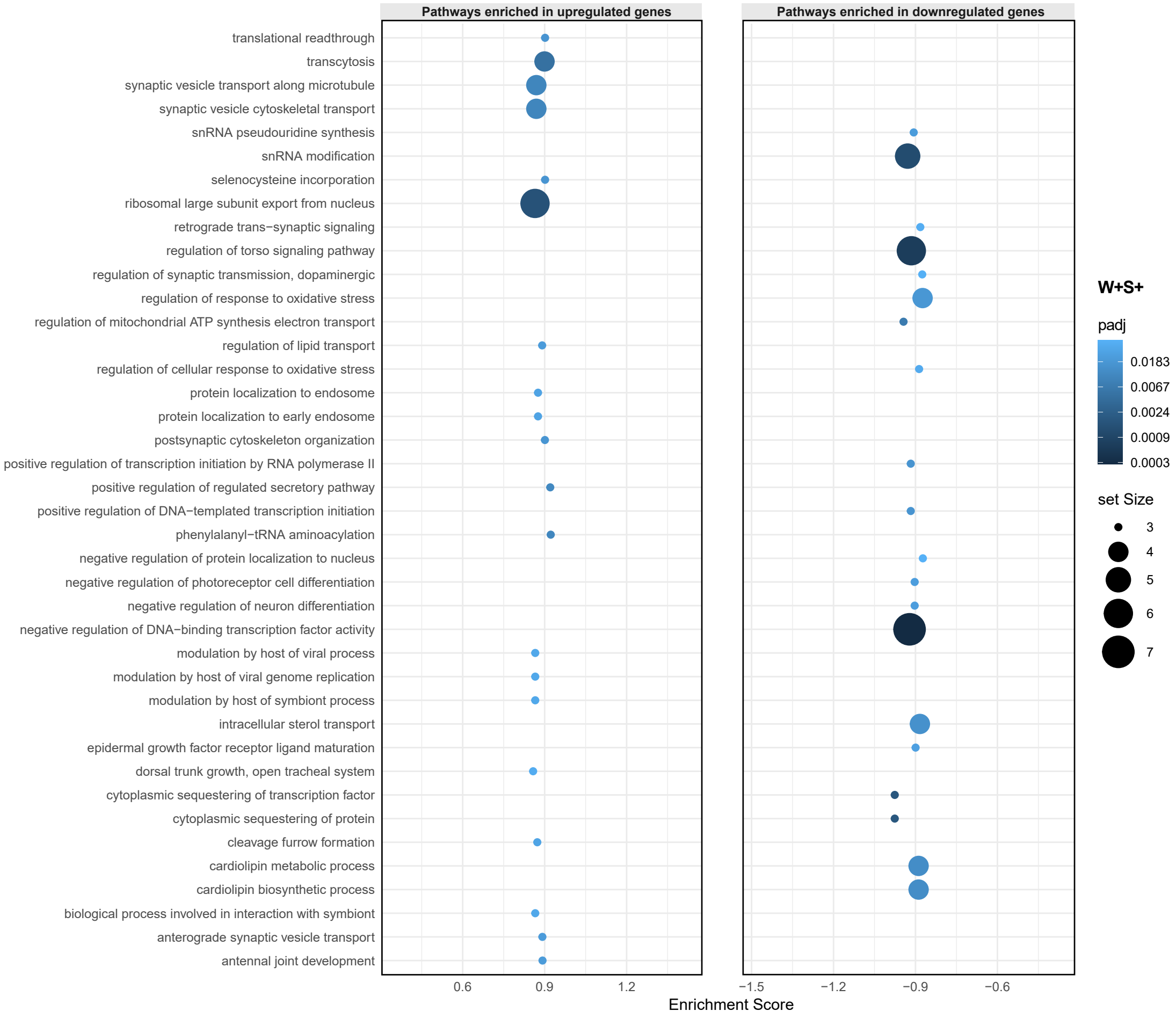

Supplement: Supplementary file 1 [file Data_Sheet_1.zip › Figure S18.pdf]

Tree scale: 1

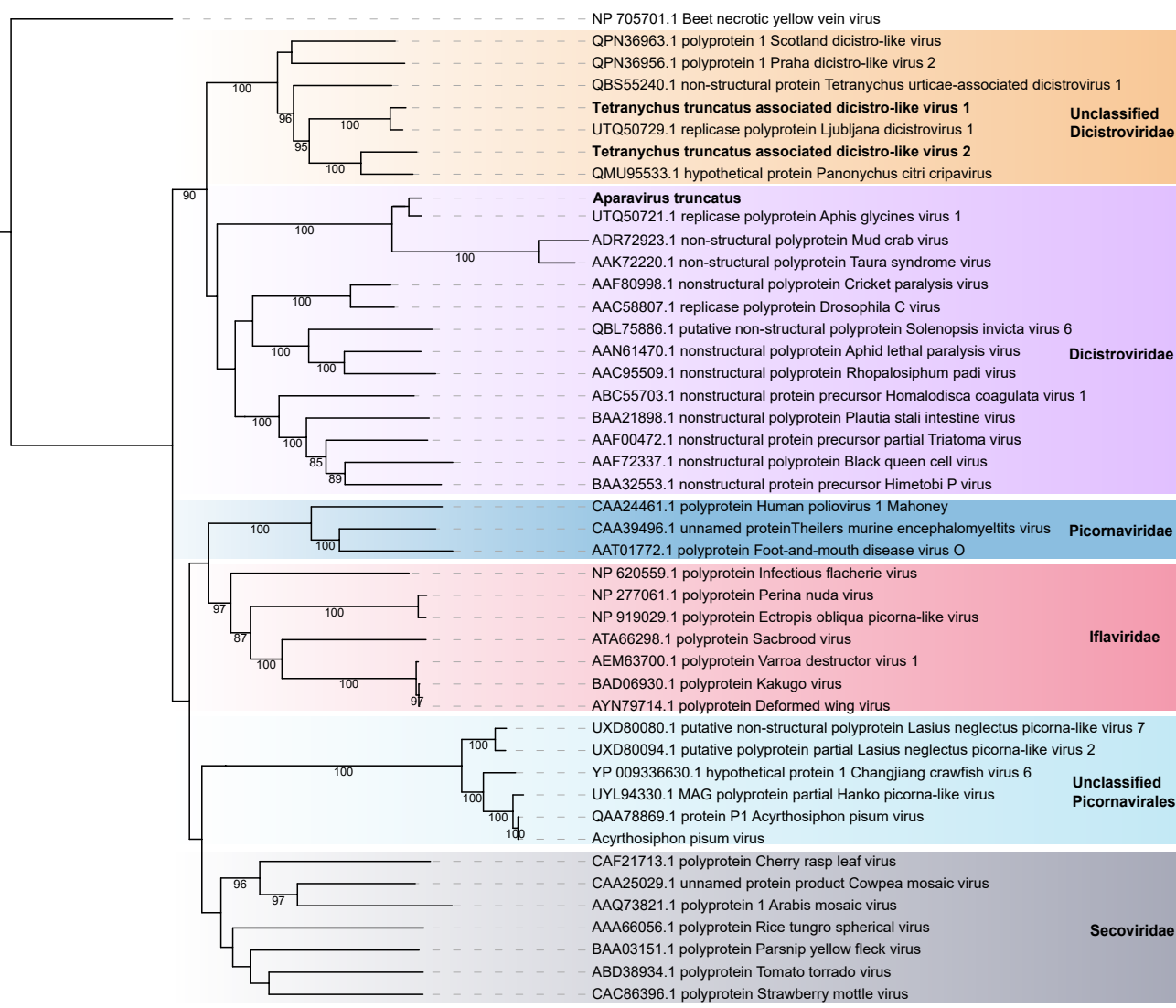

Supplement: Supplementary file 1 [file Data_Sheet_1.zip › Figure S2.pdf]

Tree scale: 1

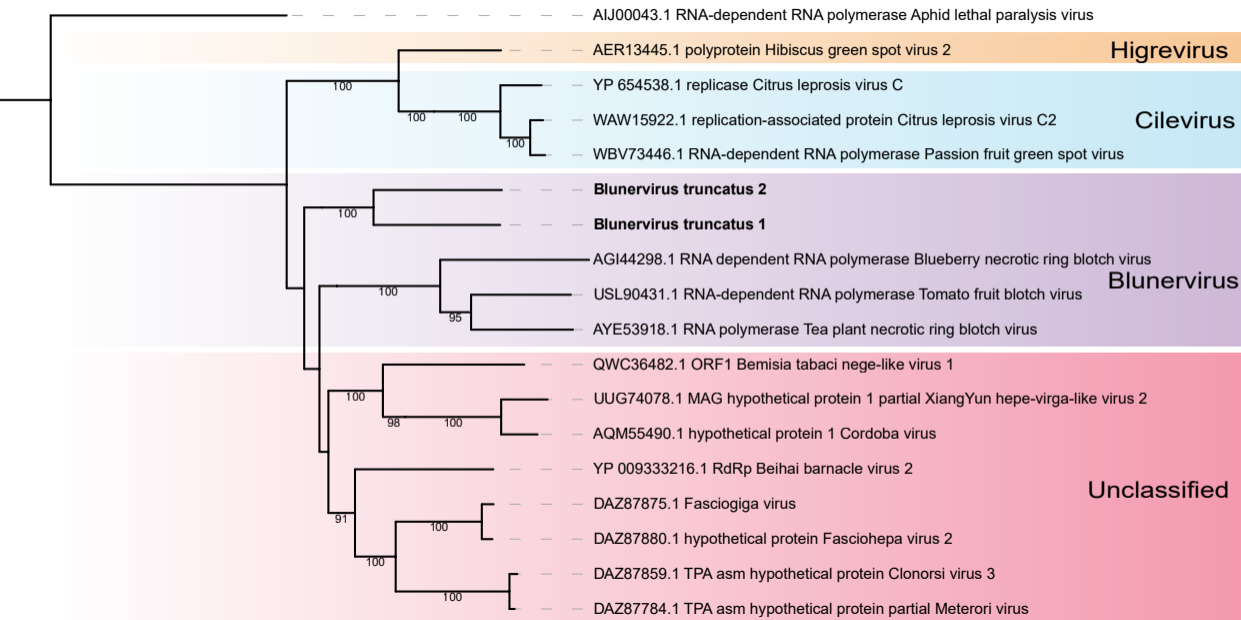

Kitaviridae

Supplement: Supplementary file 1 [file Data_Sheet_1.zip › Figure S3.pdf]

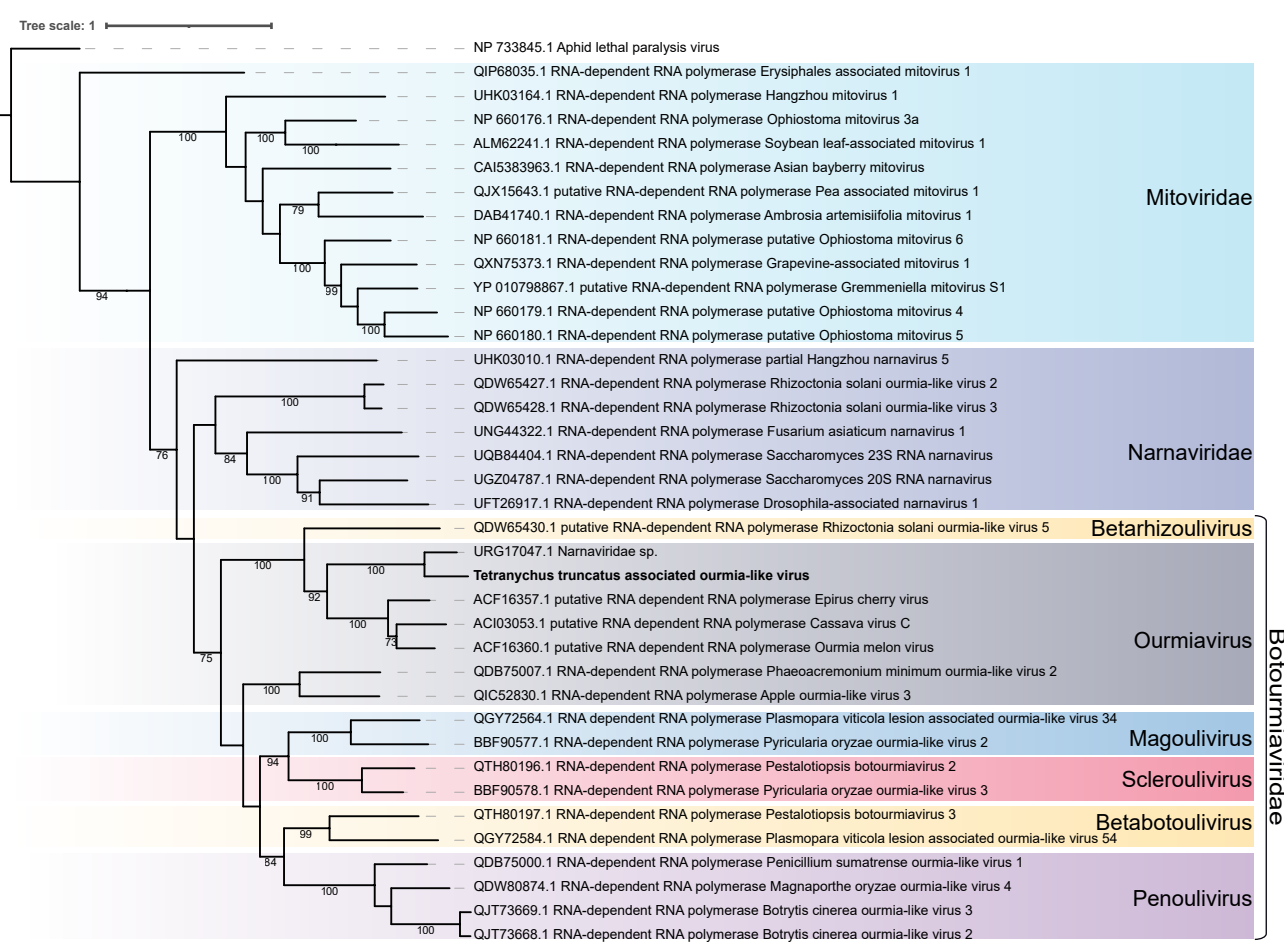

Supplement: Supplementary file 1 [file Data_Sheet_1.zip › Figure S4.pdf]

Tree scale: 1

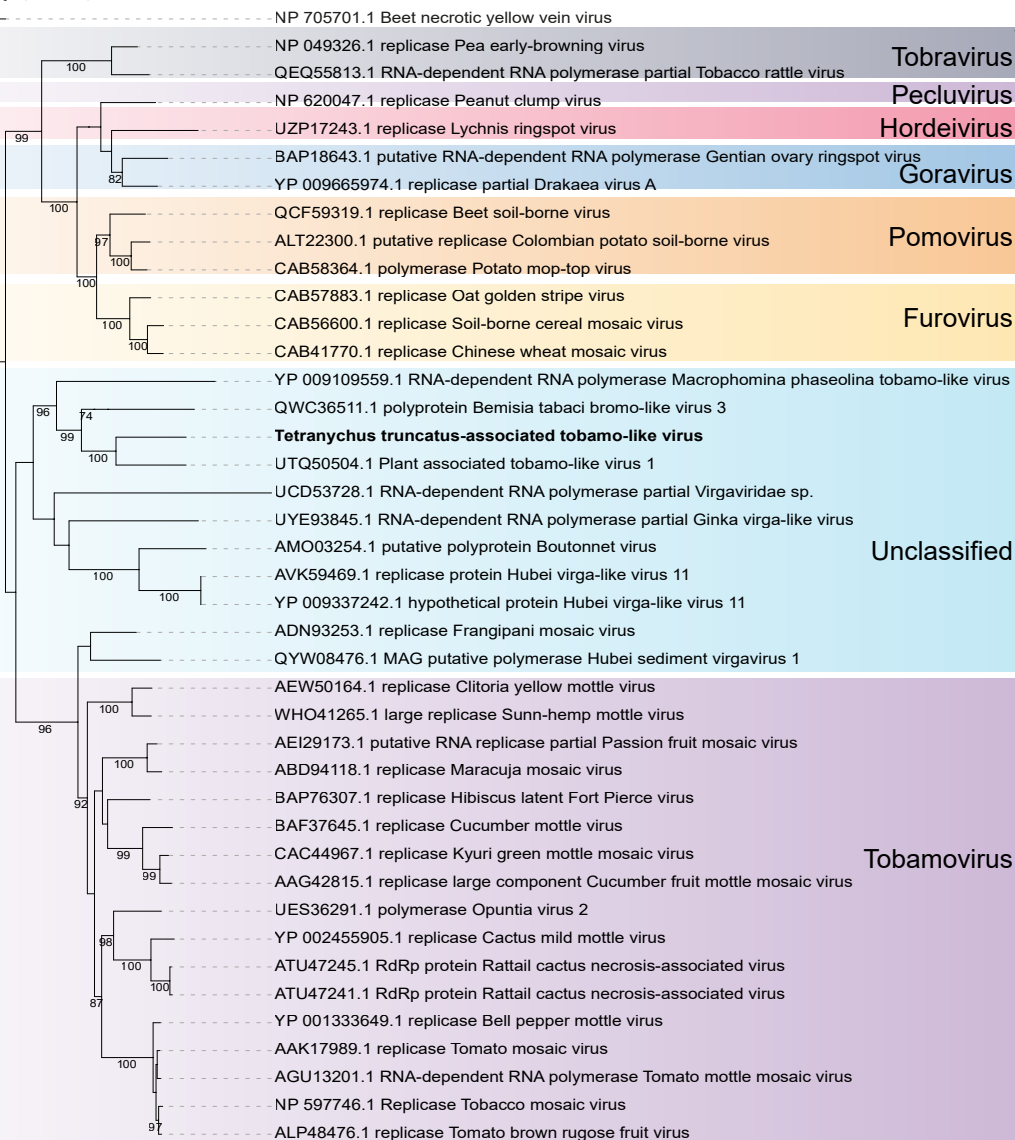

Virgaviridae

Supplement: Supplementary file 1 [file Data_Sheet_1.zip › Figure S5.pdf]

Tree scale: 1

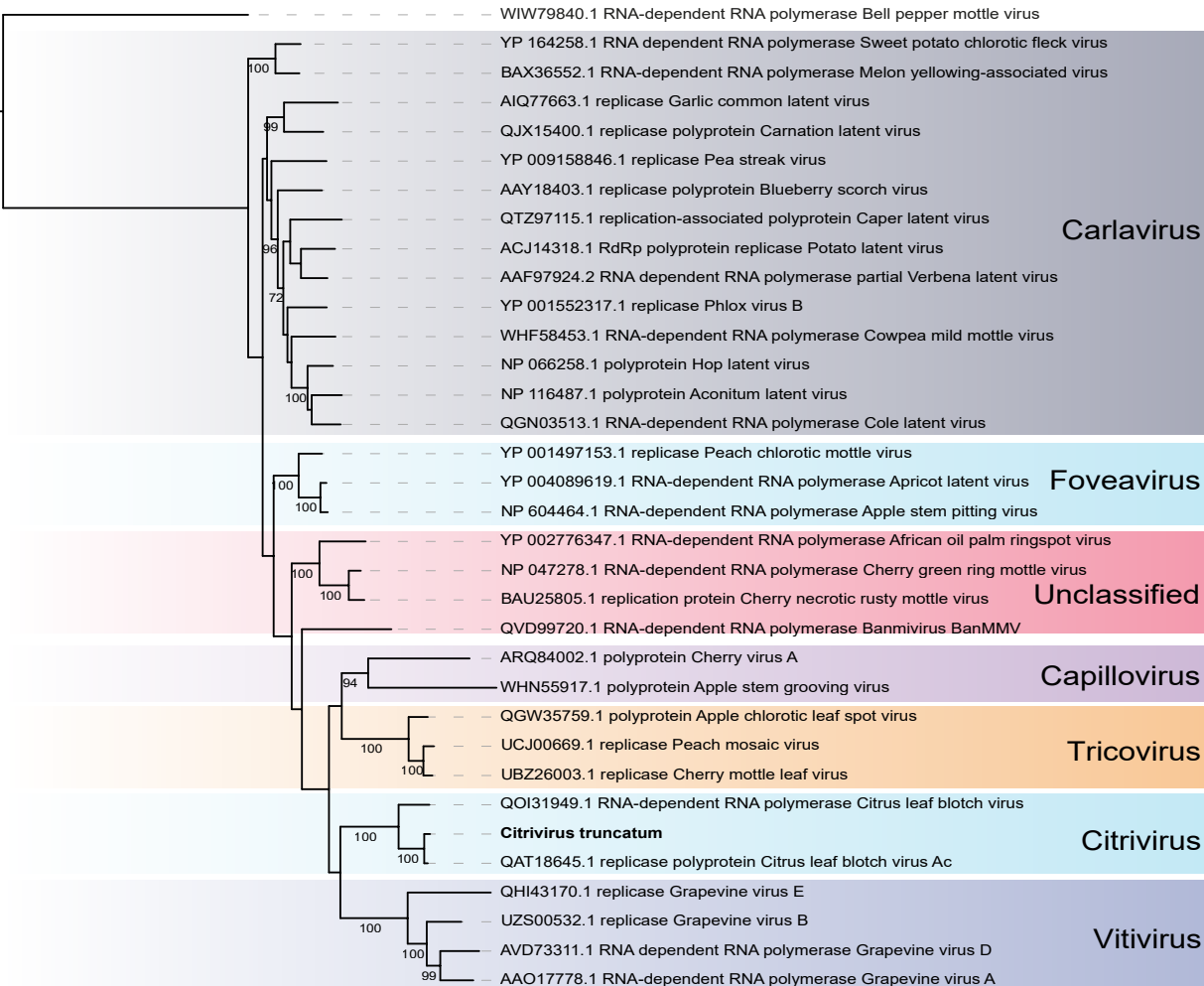

Betaflexiviridae

Supplement: Supplementary file 1 [file Data_Sheet_1.zip › Figure S6.pdf]

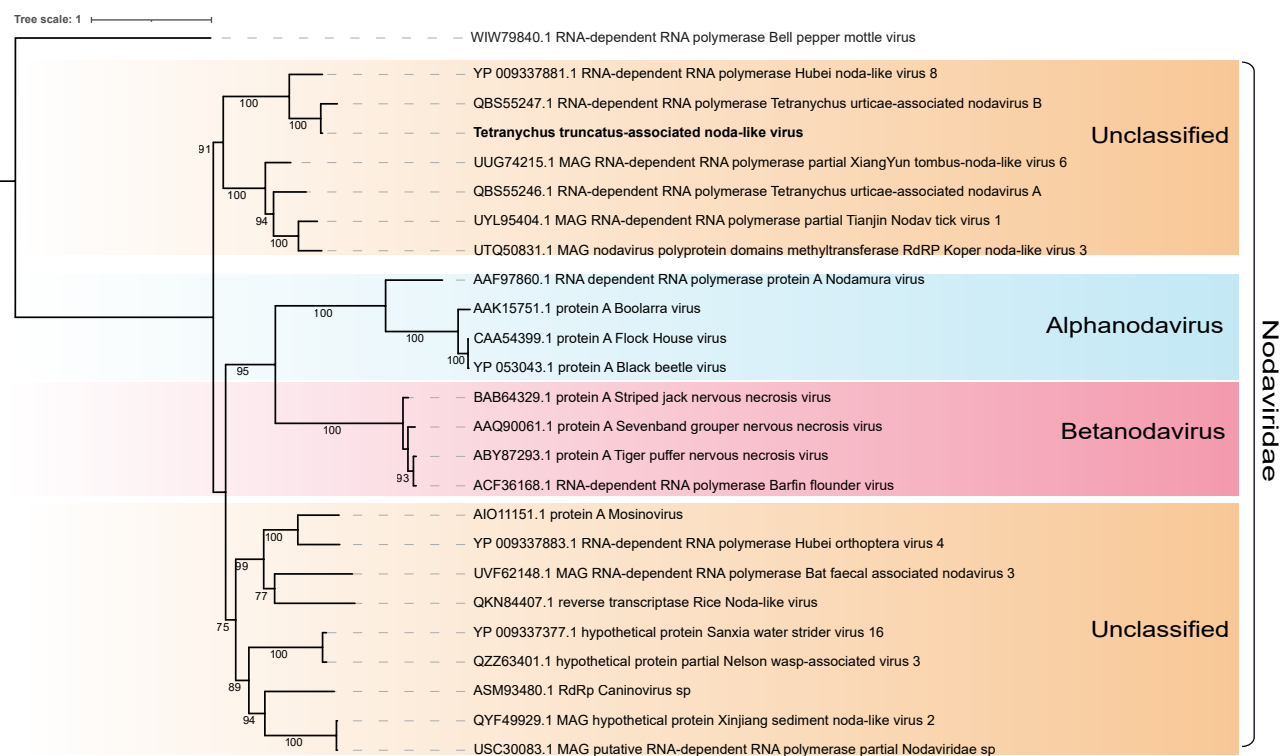

Supplement: Supplementary file 1 [file Data_Sheet_1.zip › Figure S7.pdf]

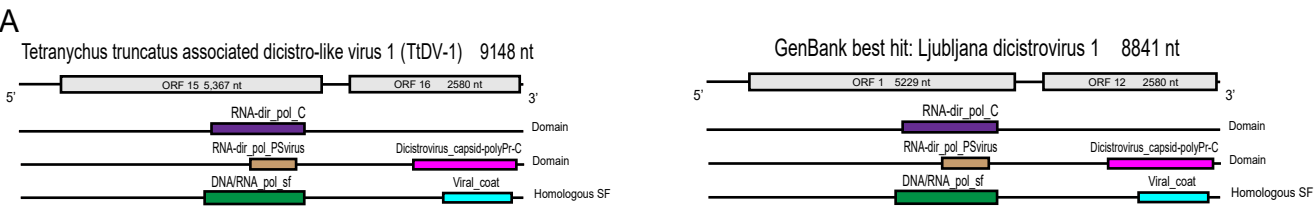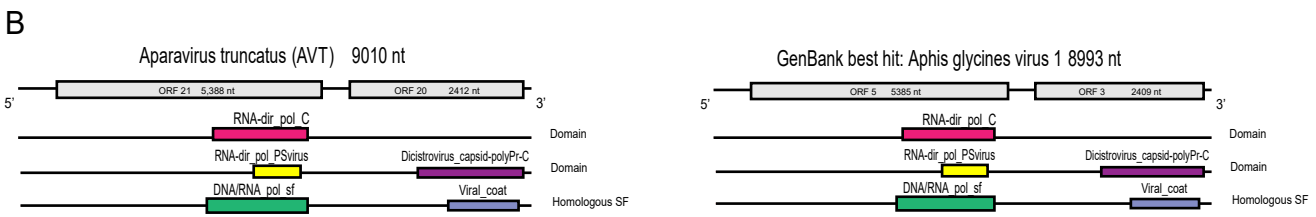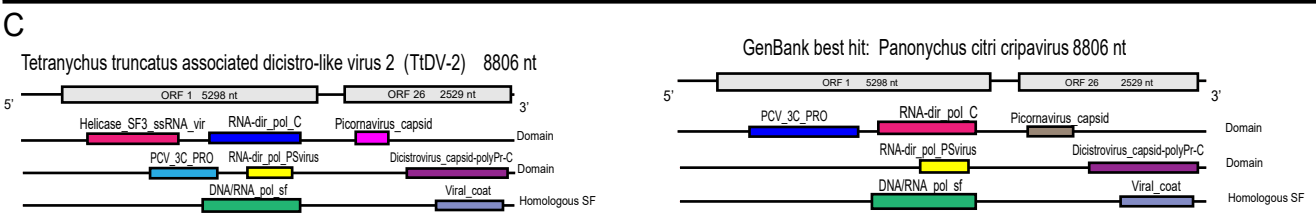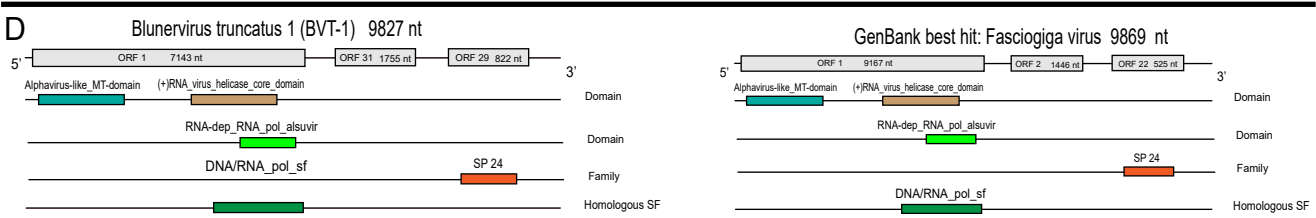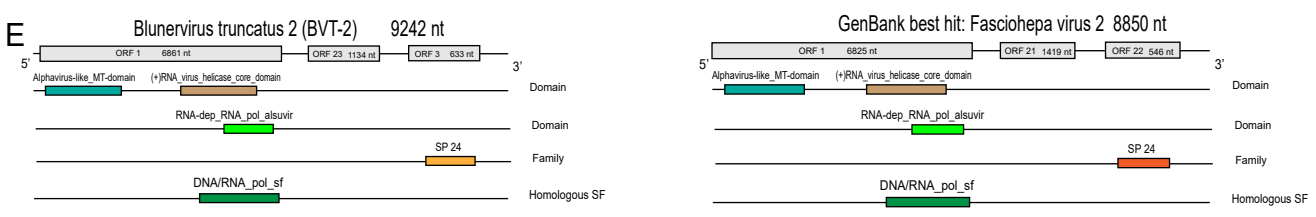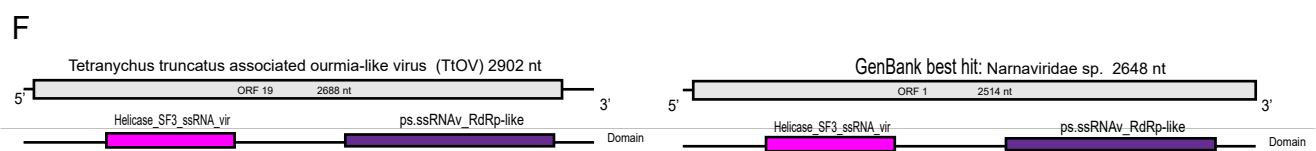

Supplement: Supplementary file 1 [file Data_Sheet_1.zip › Figure S8.pdf]
